# Ceftolozane/Tazobactam for Complex and Resistant Infections: Systematic Reviews of Comparative Efficacy Studies

**DOI:** 10.3390/antibiotics15020190

**Published:** 2026-02-09

**Authors:** Ignacio Martin-Loeches, Ryan K. Shields, Emre Yücel, Shalini Bagga, Maesumeh Korei, Hariprasad Esam, Nidhi Sharma, Carolyn Cameron

**Affiliations:** 1 Department of Intensive Care Medicine, Multidisciplinary Intensive Care Research Organization (MICRO), St. James’s Hospital, D08 NHY1 Dublin, Ireland; 2 Department of Medicine, University of Pittsburgh, Pittsburgh, PA 15260, USA; 3 Merck & Co., Inc., 126 East Lincoln Avenue, P.O. Box 2000, Rahway, NJ 07065, USA; 4 CHEORS LLC, Chalfont, PA 18914, USA; 5 MSD, Sydney 2113, Australia

**Keywords:** Ceftolozane/tazobactam, mortality, clinical cure, microbiological eradication, cUTI, cIAI, HABP/VABP

## Abstract

**Introduction:** The emergence and spread of multidrug-resistant infections has resulted in significant clinical and economic burdens. To address these infections, novel therapy combinations are needed. Ceftolozane/tazobactam is a treatment option that targets multidrug-resistant pathogens and may offer improved patient outcomes compared to traditional antibiotics that are now often ineffective. **Objectives:** Our objective was to collate findings from comparative efficacy studies to assess the efficacy of ceftolozane/tazobactam for the indications of complex intra-abdominal infection, complex urinary tract infection, ventilated hospital-acquired bacterial pneumonia and ventilator-associated bacterial pneumonia. **Methods:** Two systematic literature reviews were conducted, including randomized controlled trials comparing ceftolozane/tazobactam with other interventions for complex intra-abdominal infection, complex urinary tract infection, ventilated hospital-acquired bacterial pneumonia and ventilator-associated bacterial pneumonia indications. The outcomes of interest were mortality, clinical cure and microbiological eradication. **Results:** Ceftolozane/tazobactam was determined to be non-inferior to comparators for all outcomes of interest. All-cause mortality for ceftolozane/tazobactam displayed non-inferiority to meropenem, with the largest numerical differences in all-cause mortality displayed in susceptible patients, such as those with severe renal impairment. Similarly, the clinical cure and microbiological eradication for ceftolozane/tazobactam demonstrated non-inferiority compared to meropenem or levofloxacin. **Conclusions:** These reviews support the role of ceftolozane/tazobactam as an alternative option, particularly when MDR pathogens are suspected or documented. Their findings may contribute to the standardization of treatment guidelines, ultimately helping to reduce the clinical and economic burdens associated with these infections.

## 1. Introduction

Antimicrobial resistance (AMR) poses a significant global threat and impacts populations across every country regardless of region or income level [1]. Consequently, the World Health Organization (WHO) has reported AMR as a public health concern [1].

Common forms of healthcare-associated infections include complex intra-abdominal infection (cIAI), complex urinary tract infection (cUTI), ventilated hospital-acquired bacterial pneumonia (vHABP) and ventilator-associated bacterial pneumonia (VABP) [2]. These healthcare-associated infections, which are often associated with AMR, can be attributed to six main pathogens: *Enterococcus faecium*, *Staphylococcus aureus*, *Klebsiella pneumoniae*, *Acinetobacter baumannii*, *Pseudomonas aeruginosa*, and *Enterobacterales* spp. [3]. In particular, *P. aeruginosa* was responsible for 10.3% of HABP and hospital-acquired lower respiratory tract infections and 7.9% of healthcare-acquired infections overall in the Point Prevalence Survey of European Hospitals 2022–2023 [4]. In the US in 2017, the CDC estimated that there were 32,600 cases of *P. aeruginosa* in hospitalized patients, leading to 2700 deaths [5]. A recent study found that approximately 10% of cUTI, cIAI and HABP/VABP pathogens tested were non-susceptible to carbapenem, the typical treatment for severe, complicated infections [3]. Of these isolates, *P. aeruginosa* and *A. baumannii* were found to have the highest levels of resistance (>20%) to carbapenems [3]. A growing number of infections are caused by pathogens that are resistant to multiple antibiotics. In 2021, 1.27 million deaths globally were attributable to infections caused by antimicrobial-resistant organisms, a figure projected to rise to 8.22 million by 2050 [6]. AMR has led to an increase in healthcare resource utilization, which contributes to direct and indirect costs. Healthcare costs directly related to AMR are approximately US $66 billion per year and are expected to rise to US $159 billion per year by 2050 [7].

Due to the rise in multidrug-resistant (MDR) infections, traditional antibiotics are becoming less effective, which complicates care, leading to clinical and economic burdens, necessitating novel treatment combinations. To deal with the rising burden of AMR infections, there is a need for new antimicrobial therapies with novel mechanisms that effectively treat otherwise resistant infections. Ceftolozane/tazobactam (C/T) is a treatment made up of an antipseudomonal cephalosporin and a β-lactamase inhibitor for use against MDR or extensively drug-resistant Gram-negative pathogens such as *P. aeruginosa* and *Enterobactarales* [8]. C/T has been approved for use in patients with cIAI, cUTI and HABP/VABP and has demonstrated decreased resistance development and increased lung penetration when compared to other antipseudomonal antibiotics [8,9].

The aim of this paper was to evaluate the comparative efficacy of C/T versus standard comparators in vHABP/VABP, cIAI and cUTI based on the findings of two systematic literature reviews (SLRs).

## 2. Results

### 2.1. Overview of Included Studies

#### 2.1.1. Ventilated Hospital-Acquired Bacterial Pneumonia/Ventilator-Associated Bacterial Pneumonia

The original SLR and the SLR update yielded a total of 2592 publications from Medline, EMBASE, and Cochrane databases. After excluding 380 duplicates, a total of 2212 publications underwent title and abstract screening, with 142 publications found to be eligible for full-text review. After full-text reading, eight publications were included. Hand searches were also performed from other sources (e.g., conference proceedings, clinicaltrials.gov, and bibliographic searching), and one conference abstract was included.

**Figure 1:**
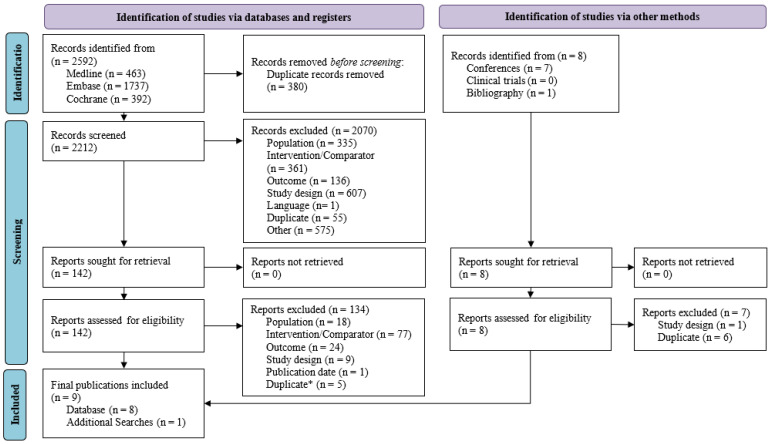
PRISMA study selection flow diagram from the SLR on ventilated hospital-acquired bacterial pneumonia/ventilator-associated bacterial pneumonia. * One record was included in the original SLR: a trial registry website. All the data published in the trial registry have been published in the publications included during the SLR update. The registry record has been excluded from the PRISMA as a duplicate.

In total, nine publications from one unique trial, ASPECT-NP, were included (Figure 1). Study characteristics are outlined in Table 1. ASPECT-NP included patients with VABP and vHABP, a subset of HABP which requires mechanical ventilation.

#### 2.1.2. Complex Intra-Abdominal Infection and Complex Urinary Tract Infection

A total of 1173 publications were identified from PubMed, EMBASE, and Cochrane databases. After excluding 226 duplicates, a total of 947 publications were included for title and abstract screening, resulting in the identification of 87 publications eligible for full-text review. After full-text reading, six publications were included. Hand searches were also performed from other sources (e.g., conference proceedings, clinicaltrials.gov, and bibliographic searching), and one conference abstract was included.

**Figure 2:**
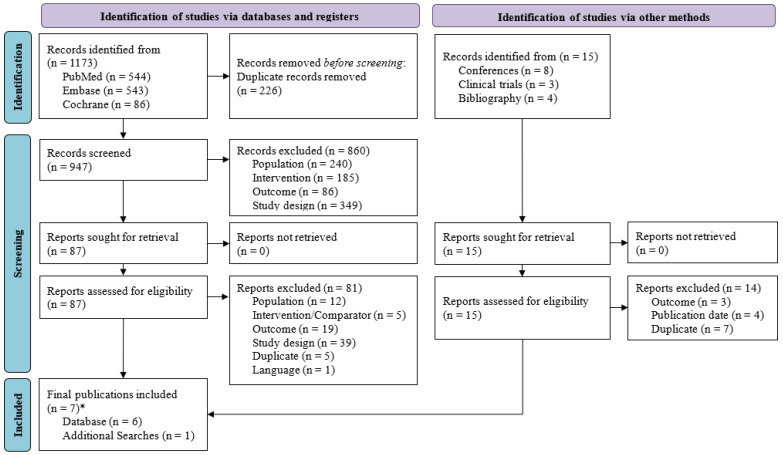
PRISMA study selection flow diagram from the SLR on complex intra-abdominal infection and complex urinary tract infection. * Seven studies included from four unique trials.

In total, seven publications from four unique trials (ASPECT-cIAI, ASPECT-cUTI, NCT01147640 and NCT03830333) were included (Figure 2). Study characteristics for the included studies can be found in Table 2.

### 2.2. All-Cause Mortality

#### 2.2.1. Ventilated Hospital-Acquired Bacterial Pneumonia/Ventilator-Associated Bacterial Pneumonia

All-cause mortality (ACM) was reported for all publications on the ASPECT-NP trial (Table 3). In the overall trial population at 28 days, ACM was similar in the C/T group (24%) compared to the meropenem group (25.3%) for the whole study population with vHABP/VABP [10]. Across the different subgroups analyzed within this population, C/T demonstrated numerically lower or similar mortality rates compared to meropenem (Table S14). The overall mortality was numerically lower in several subgroups treated with C/T compared to meropenem, including vulnerable patients such as those with severe renal impairment (RI) (30.8% versus 57.1%), extended spectrum beta-lactamase (ESBL) positive infections (8.0% versus 30.3%), or failure of initial therapy (22.6% versus 45.0%) [15,16,17]. C/T demonstrated a non-inferiority to meropenem across all subgroups.

#### 2.2.2. Complex Intra-Abdominal Infection and Complex Urinary Tract Infection

Three publications reported ACM in patient populations with cIAI from ASPECT-cIAI, NCT01147640 and NCT03830333. ACM was low across all three trials, and no statistically significant differences were identified between C/T plus metronidazole versus meropenem (Table 4) [19,21,22].

No publications reported ACM for patients with cUTI.

### 2.3. Clinical Cure

#### 2.3.1. Ventilated Hospital-Acquired Bacterial Pneumonia/Ventilator-Associated Bacterial Pneumonia

Eight publications reported the clinical cure attributed to C/T compared to meropenem. Similar or favorable clinical results were presented across all publications. The rate of clinical cure was similar in the C/T group (54.4%) compared to the meropenem group (53.3%) for the whole study population with vHABP/VABP [10]. C/T demonstrated a non-inferiority to meropenem across all subgroups (Table 5).

Subgroup analyses demonstrated that patients in the clinically evaluable populations with vHABP/VABP who had failed initial anti-bacterial therapy had numerically higher rates of clinical cure when C/T was utilized compared to meropenem (63.6% vs. 45.0%; Table S15) [16]. Additionally, use of C/T in patients with ESBL-positive infections and patients with Gram-negative infections resulted in numerically higher rates of clinical cure when compared to meropenem (72.0% vs. 66.7% and 75.2% vs. 66.7%, respectively) [11,17].

#### 2.3.2. Complex Intra-Abdominal Infection and Complex Urinary Tract Infection

Three publications reported on clinical cure for cIAI from the ASPECT-cIAI trial. The rates of clinical cure were similar in the C/T plus metronidazole group compared to the meropenem group in all three publications investigating cIAI (Table 6) [19,21,22]. C/T plus metronidazole demonstrated a non-inferiority to meropenem across all subgroups (Table S16).

Two studies reported on clinical cure in patients with cUTI based on ASPECT-cUTI. The rate of clinical cure was numerically higher in the C/T group (92%) compared to the levofloxacin group (88.6%) [23]. C/T demonstrated non-inferiority to levofloxacin across all subgroups (Table 7). In the post hoc analysis of ASPECT-cUTI by Huntington (2016) differentiating patients with cUTI and pyelonephritis, C/T was identified as non-inferior when compared to levofloxacin [24].

### 2.4. Microbiological Eradication

#### 2.4.1. Ventilated Hospital-Acquired Bacterial Pneumonia/Ventilator-Associated Bacterial Pneumonia

Eight publications reported on microbiological eradication in patients with vHABP/VABP based on ASPECT-NP. The rate of microbiological eradication was numerically higher in the C/T group (73.1%) compared to the meropenem group (68.0%) for the whole study population with vHABP/VABP (Table 8) [10]. C/T demonstrated a non-inferiority to meropenem across all subgroups (Table S17). Notably, subgroup analyses of vulnerable patient populations such as those with severe RI or patients requiring mechanical ventilation treated with C/T displayed numerically higher microbiological eradication when compared to meropenem (69.2% vs. 57.1% and 78.2% vs. 62.0%, respectively) [12,15].

#### 2.4.2. Complex Intra-Abdominal Infection and Complex Urinary Tract Infection

Two publications reported on microbiological eradication in patients with cUTI. The rate of microbiological eradication was numerically higher in the C/T group (80.4%) compared to the levofloxacin group (72.1%) for the whole study population [23]. This 8.3% difference is statistically significant (95% confidence interval 2.4–14.1%) [23]. C/T demonstrated a non-inferiority to levofloxacin in patients with cUTI (Table 9) [23,24]. Microbiological eradication was not reported in patients with cIAI.

### 2.5. Risk of Bias

#### 2.5.1. Ventilated Hospital-Acquired Bacterial Pneumonia/Ventilator-Associated Bacterial Pneumonia Systematic Literature Review

**Figure 3:**
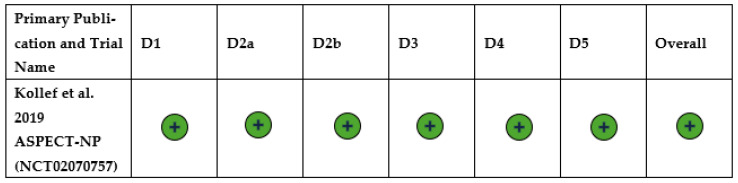
Risk of bias summary assessment for the systematic literature review on ventilated hospital-acquired bacterial pneumonia/ventilator-associated bacterial pneumonia [10]. Abbreviations: D1: Domain 1; D2a: Domain 2a; D2b: Domain 2b; D3: Domain 3; D4: Domain 4; D5: Domain 5. Note: Meaning of results: 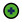 Low risk of bias. D1: Risk of bias arising from the randomization process. D2a: Risk of bias due to deviations from the intended interventions (effect of assignment to intervention). D2b: Risk of bias due to deviations from the intended interventions (effect of adhering to intervention). D3: Risk of bias due to missing outcome data. D4: Risk of bias in measurement of the outcome. D5: Risk of bias in selection of the reported result.

The ASPECT-NP trial had low risk of bias across all domains, including risk of bias arising from the randomization process, risk of bias due to deviations from the intended interventions (effect of assignment to intervention), risk of bias due to deviations from the intended interventions (effect of adhering to intervention), risk of bias due to missing outcome data, risk of bias in the measurement of the outcome, and risk of bias in the selection of the reported result (Figure 3) [10].

#### 2.5.2. Complex Intra-Abdominal Infection and Complex Urinary Tract Infection Systematic Literature Review

**Figure 4:**
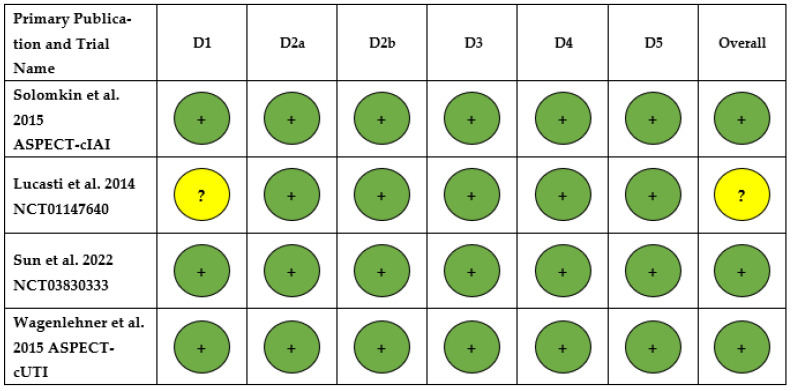
Risk of bias summary assessment for the systematic literature review on complex intra-abdominal infection and complex urinary tract infection [19,21,22,23]. Abbreviations: D1: Domain 1; D2a: Domain 2a; D2b: Domain 2b; D3: Domain 3; D4: Domain 4; D5: Domain 5. Note: Meaning of results: 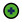 Low risk of bias. 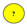: Unclear risk of bias. D1: Risk of bias arising from the randomization process. D2a: Risk of bias due to deviations from the intended interventions (effect of assignment to intervention). D2b: Risk of bias due to deviations from the intended interventions (effect of adhering to intervention). D3: Risk of bias due to missing outcome data. D4: Risk of bias in measurement of the outcome. D5: Risk of bias in selection of the reported result.

Overall, the ASPECT-cIAI trial, ASPECT-cUTI trial and NCT03830333 trail had a low risk of bias for all domains, including risk of bias arising from the randomization process, risk of bias due to deviations from the intended interventions (effect of assignment to intervention), risk of bias due to deviations from the intended interventions (effect of adhering to intervention), risk of bias due to missing outcome data, risk of bias in the measurement of the outcome, and risk of bias in the selection of the reported result [22,23,24]. The NCT01147640 trial had an unclear risk of bias arising from the randomization process and risk of bias in the selection of the reported result (Figure 4) [21].

## 3. Discussion

These SLRs evaluated the efficacy of C/T for the treatment of HABP/VABP and cIAI and cUTI based on data from RCTs. Most studies demonstrated the non-inferiority of C/T to standard comparators, supporting its role as an effective treatment option for these indications.

Nine publications were included for HABP/VABP, all of which were based on ASPECT-NP reporting on at least two of the outcomes of interest; ACM, clinical cure and microbiological eradication. Seven publications from four unique trials were included for cIAI and cUTI (ASPECT-cIAI, ASPECT-cUTI, NCT01147640 and NCT03830333). Three trials reported on ACM (ASPECT-cIAI, NCT01147640 and NCT03830333) and two trials reported on clinical cure (ASPECT-cIAI, ASPECT-cUTI), while only one trial reported on microbiological eradication (ASPECT-cUTI).

Overall, ACM at 28 days was non-inferior with C/T when compared to meropenem (24.0% versus 25.3%) in patients with vHABP/VABP [16]. Notably, this review reported evidence that C/T demonstrated numerically lower mortality, consistent with non-inferiority but not powered to detect superiority, when compared to meropenem in patients with severe RI (30.8% vs. 57.1%), who are known to have worse infection outcomes than the general population. Furthermore, C/T was associated with numerically lower ACM versus meropenem in patients with ESBL-producing *Enterobacterales* (13.0% vs. 29.4%) and patients who failed initial therapy (22.6% vs. 45.0%), indicating that C/T may provide an alternative treatment option in vulnerable/high-risk patient populations [11,15,16,25]. Similarly, ACM was low across all three trials investigating C/T for the treatment of cIAI, displaying a non-inferiority to meropenem. No studies including cUTI reported on ACM. These results are similar to those found in studies external to these SLRs. For example, SPECTRA was a large, multinational, multicenter trial which found that C/T was associated with low ACM in patients with Gram-negative infections in the real world. Similarly, a 2024 retrospective, observational study compared the outcomes of non-COVID-19 hospitalized patients with pneumonia caused by MDR *P. aeruginosa* and demonstrated non-inferior rates of mortality of C/T when compared to ceftazidime/avibactam (C/A) [26,27].

Within this SLR, in ASPECT-NP, C/T was non-inferior to meropenem when considering clinical cure in patients with vHABP/VABP. Similarly, C/T was found to be non-inferior to levofloxacin in patients with cUTI, while C/T plus metronidazole was non-inferior to meropenem for the treatment of cIAI when considering clinical cure. These results suggest that C/T plus metronidazole or C/T alone may be considered an effective treatment choice in patients with vHABP/VABP, cIAI and cUTI. These results are further externally validated by CACTUS, a multicenter, retrospective study which demonstrated the non-inferiority of C/T to ceftazidime/avibactam (C/A) in patients with MDR *P. aeruginosa*. C/T displayed non-inferiority in clinical cure when compared with C/A [28]. Notably, in this study, patients who had previously failed initial therapies displayed higher rates of clinical cure when C/T was administered compared with meropenem (63.6% vs. 45.0%) [16]. These findings highlight the potential efficacy of C/T in treating patients for whom other antimicrobials are no longer effective.

Microbiological eradication was investigated in ASPECT-NP, displaying non-inferiority when C/T was compared to meropenem. Within this trial, vulnerable patient populations such as patients with severe RI or patients requiring mechanical ventilation showed numerically higher microbiological eradication with C/T versus meropenem (69.2% vs. 57.1% and 78.2% vs. 62.0%, respectively) [12,15]. Additionally, within the ASPECT-cUTI trial, C/T demonstrated a non-inferiority to levofloxacin when considering microbiological eradication. These findings are similar to the results from an external review conducted by Lizza (2021) investigating the safety and efficacy of C/T for Gram-negative infections, which found that C/T was associated with similar levels of microbiological eradication (81%) [29].

Outside of these SLRs, the appropriateness of C/T as an alternative treatment option has been demonstrated by the higher rates of susceptibility in *P. aeruginosa* isolates (97.5%) when compared with meropenem (76.0%) [30]. In addition, C/T has been suggested to provide a good activity against AmpC-producing *P. aeruginosa* and may be less affected by efflux-related resistance mechanisms [31,32]. When considered alongside the suggested non-inferiority to other antimicrobials in this study, the use of C/T may result in better coverage in high-risk patients. However in order to mitigate the risk of C/T resistance, stewardship programs, including the development of treatment guidelines, the education of clinicians, and antimicrobial cycling are key [33].

These findings indicate that C/T may be an effective alternative treatment to meropenem and levofloxacin and may be appropriate to consider when alternative treatments are limited by AMR or other factors, such as allergy or intolerance.

Given the demonstrated non-inferiority of C/T versus meropenem for cIAI and HABP/VABP and versus levofloxacin for cUTI, C/T should be positioned as a targeted reserve agent rather than as a first-line therapy. Antimicrobial stewardship principles support the use of C/T, assuming it is guided by local susceptibility patterns and institutional antibiogram, particularly in settings with a high prevalence of MDR *P. aeruginosa* or ESBL-producing Enterobacterales, or following the failure of prior therapy, alongside robust microbiology support such as rapid diagnostics and timely susceptibility testing to confirm pathogens, characterize resistance mechanisms, and enable de-escalation when feasible. Where available, indication-specific dosing with renal adjustment and therapeutic drug monitoring is recommended, particularly in vulnerable patient populations. Furthermore, clear criteria for escalation, such as documented or strongly suspected resistance to standard antipseudomonal agents or intolerance to comparators, and reassessment at 48–72 h for step-down or discontinuation are essential. The sensible, protocolized use of C/T may reduce the selective pressure relative to broad empiric carbapenem use and help to preserve carbapenem activity; however, it should be noted that resistance can still emerge. Mitigation strategies for resistance development include preauthorization or prospective audit and feedback, the use of shortest effective treatment durations, the avoidance of unnecessary combination therapy, and the ongoing local surveillance of C/T susceptibility trends.

## 4. Materials and Methods

Two SLRs were conducted. One SLR included RCTs evaluating the relative efficacy of C/T in adult patients with HABP/VABP, while the second SLR focused on the literature where C/T was utilized in patients with cIAI and cUTI. Both SLRs were conducted in line with Preferred Reporting Items for Systematic reviews and Meta-Analyses (PRISMA) guidelines. The PRISMA checklist can be seen in Table S18.

### 4.1. Study Eligibility and Data Sources

Study eligibility criteria were defined in the population, interventions, comparisons, outcomes, time, and study design (PICOTS) structure outlined in Tables S1 and S2. The data sources are outlined in Table S3. Systematic searches were conducted in PubMed/Medline (Ovid), Embase (Ovid), and Cochrane databases from inception up to July 2024. Relevant conference proceedings from 2018 to 2024, clinicaltrials.gov and bibliographies of identified systematic reviews were also hand-searched (Tables S4–S13).

### 4.2. Literature Search and Screening Process

Searches for the SLR looking at HABP/VABP were conducted on 27 September 2018, and an update to the SLR was carried out with searches conducted on 18 July 2024. Searches for the SLR on cIAI and cUTI were carried out on 18 July 2024.

The studies identified by the searches were screened using a two-stage process. In the first stage, titles and abstracts were independently screened by two researchers to determine eligibility. Disagreements were resolved by a third reviewer. In the second stage, full-text articles of potentially relevant studies were reviewed by two independent researchers to confirm eligibility. Conflicts were resolved by a third reviewer. In this manuscript, we will report the following outcomes: all-cause mortality (ACM), clinical cure, and microbiological eradication. Additional outcomes were specified in the PICOTS for the SLRs (Tables S1 and S2).

The quality of individual trials included was assessed using the revised Cochrane risk of bias assessment tool [34].

### 4.3. Outcomes and Definitions

The definition of clinical cure was similar across all RCTs included in the SLRs, defined as the alleviation of signs and symptoms from baseline. Comparably, a favorable microbiological response was defined as the eradication of baseline pathogens across all RCTs. Each definition included slight variations in these based on infection type. For example, a microbiological eradication in HABP/VABP was defined as a ≥1 log reduction in baseline LRT pathogen bacterial burden, with a resulting per-pathogen count of ≤104 cfu/mL for endotracheal aspirate or sputum, ≤103 cfu/mL for bronchoalveolar lavage, or ≤102 cfu/mL for protected brush specimen.

## 5. Conclusions

The evidence identified by these SLRs highlights the potential for C/T to be used in patients with cIAI, cUTI and HABP/VABP to treat a range of Gram-negative infections, including those caused by MDR *P. aeruginosa*. This evidence was strongest in non-inferiority RCTs, with a descriptive analysis on RWE offering more limited support and lower-quality data on the use of C/T in MDR infections specifically. These SLRs may inform guideline discussions and local stewardship decisions, which could facilitate the more effective treatment of complex MDR infections, therefore reducing the clinical and economic impacts associated with these infections.

## 6. Limitations

The SLR for HABP/VABP relies solely on data from the ASPECT-NP trial which reduces the generalizability of the findings and introduces potential biases related to participant selection and outcome reporting. Due to the low numbers, a meta-analysis could not be performed, and heterogeneity could not be quantified, limiting the confidence in the findings.

Additionally, the SLR for cUTI and cIAI included trials which were designed to test noninferiority, so the sample size was calculated accordingly and was not adequately powered to test comparative effectiveness. For instance, in the publication by Lucasti et al. (2014), a small number of patients is included, and the patients are not equally randomized [21]. Furthermore, within the publication by Sun et al. (2022), differences in the clinical response rates in the ITT population were influenced by missing or intermediate responses in the C/T plus metronidazole arm compared to the meropenem arm [22].

A notable proportion of the results included in these SLRs came from post hoc or subgroup analyses (e.g., renal impairment, ESBL-positive infections), which are exploratory and not pre-specified in the original trial designs. These analyses may be prone to type I errors, so the results should be interpreted with caution.

The outcomes of interest in these SLRs focused on efficacy endpoints only, and the analysis did not include safety and tolerability. While this SLR suggests that C/T may be an effective treatment alternative, conclusions cannot be drawn on safety and tolerability, which are important factors when considering treatment guidelines.

The RCTs included in these SLRs largely include susceptible or non-carbapenem-resistant isolates. These results therefore may have limited generalizability to settings and infections where treatment choices or patient characteristics differ markedly from the included studies. The true MDR data included in the discussion come mainly from observational studies, which should be interpreted with caution due to possible confounding and bias.

SLRs may also introduce a potential bias from the included studies. The limitations of the SLRs highlight the need for further research, including head-to-head trials, to better understand the comparative effectiveness of C/T across diverse patient populations.

## Figures and Tables

**Table 1:** Study characteristics of included studies: Ventilated hospital-acquired bacterial pneumonia/ventilator-associated bacterial pneumonia (ASPECT-NP).

Study (Year)	Primary or Secondary Publication	Patient Enrollment Time	No. of Sites/Centers	Study Design	Selection Criteria for Patients
**Kollef** **(2019) [10]**	Primary	16 January 2015 and 27 April 2018	263 hospitals across 34 countries	RCT, Phase 3, double-blind, non-inferiority, multicenter trial	Patients ≥18 years intubated and mechanically ventilated, and had vHABP/VABP
**Martin-Loeches (2022) [11]**	Secondary			Subgroup analysis of ASPECT-NP trial	Patients with LRT pathogens
**Timsit** **(2021) [12]**	Secondary			Subgroup analysis of ASPECT-NP trial	Patients with vHABP
**Martin-Loeches (2023) [13]**	Secondary			Post hoc analysis of ASPECT-NP trial	Patients with respiratory or cardiovascular dysfunction
**Shorr** **(2021) [14]**	Secondary			Post hoc analysis of ASPECT-NP trial	Patients with augmented renal clearance
**Huntington (2020) [15]**	Secondary			Subgroup analysis of ASPECT-NP trial	Patients with renal impairment
**Kollef** **(2022) [16]**	Secondary			Post hoc analysis of ASPECT-NP trial	Patients with failure of initial anti-bacterial therapy
**Paterson (2022) [17]**	Secondary			Retrospective post hoc analysis of the ASPECT-NP trial	Patients with ESBL-producing Enterobacterales
**Martin-Loeches (2019) [18]**	Secondary			Subgroup analysis of ASPECT-NP trial	Patients with confirmed baseline LRT pathogen status

Abbreviation: ESBL: extended spectrum beta-lactamase; LRT: lower respiratory tract pathogen; RCT: randomized control trial; VABP: ventilator-associated bacterial pneumonia; vHABP: ventilated hospital-acquired bacterial pneumonia.

**Table 2:** Study characteristics of included studies: Complex intra-abdominal infection and complex urinary tract infection.

Study	Primary or Secondary	Patient Enrollment Time	No. Sites/Centers	Study Design	Selection Criteria for Patients
**ASPECT-cIAI**					
**Solomkin (2015) [19]**	Primary	December 2011–October 2013	15 countries across 61 locations	Phase 3, double-blind, RCT	Adults with clinical evidence of cIAI
**Miller et al. 2016 [20]**	Secondary		15 countries across 61 locations	Subgroup analysis: Patients with or without *P. aeruginosa*	
**Miller et al. 2015 [19]**	Secondary		Europe, number of sites NR	Subgroup analysis: European patients	European adults with cIAI
**NCT01147640**					
**Lucasti (2014) [21]**	Primary	June 2010–March 2011	5 countries across 35 sites	Phase 2, double-blind, RCT	Hospitalized male and female patients with evidence of cIAI requiring surgical intervention
**NCT03830333**					
**Sun (2022) [22]**	Primary	March 2019–October 2020	China in 21 sites across 14 provinces	Phase 3, double-blind, RCT	Chinese descents with a diagnosis of cIAI
**ASPECT-cUTI**					
**Wagenlehner (2015) [23]**	Primary	June 2011–September 2013	25 countries across 209 centers	Phase 3, double-blind, double-dummy, non-inferiority, RCT	Hospital inpatients who had pyuria and a diagnosis of a cUTI or pyelonephritis
**Huntington (2016) [24]**	Secondary		25 countries across 209 centers	Subgroup analysis: cUTIs caused by levofloxacin-resistant pathogens	Hospitalized adults with pyuria and clinical signs and/or symptoms of cUTI or pyelonephritis

Abbreviations: cIAI: complicated intra-abdominal infection; cUTI: complicated urinary tract infection; RCT: randomized control trial.

**Table 3:** All-cause mortality with C/T versus meropenem in patients with ventilated hospital-acquired bacterial pneumonia/ventilator-associated bacterial pneumonia (ASPECT-NP).

Study (Year)	Outcome	C/T (% [n/N])	Meropenem (% [n/N])	Percent Difference, % (95% CI)
**Kollef (2019) [10]**	**vHABP/VABP (ITT) ^a^**			
	28-day ACM	24.0 (87/362)	25.3 (92/364)	1.1 (−5.1 to 7.4)
	**VABP (ITT) ^a^**			
	28-day ACM	24.0 (63/263)	20.3 (52/256)	–3.6 (–10.7 to 3.5)
	**HABP (ITT) ^a^**			
	28-day ACM	24.2 (24/99)	37.0 (40/108)	12.8 (0.2 to 24.8)
**Timsit (2021) [12]**	**vHABP (mITT) ^b^**			
	28-day ACM	18.2 (10/55)	36.6 (26/71)	18.4 (2.5 to 32.5)

Abbreviations: CI: confidence interval; ITT: intention-to-treat; mITT: microbiological intention-to-treat population; VABP: ventilator-associated bacterial pneumonia; vHABP: ventilated hospital-associated bacterial pneumonia. ^a^ ITT was defined as all randomized participants, regardless of whether they received study drug. ^b^ mITT was defined as the ITT participants who received ≥1 dose of study treatment and with ≥1 Gram-negative or streptococcal respiratory pathogen from baseline lower respiratory tract (LRT) cultures confirmed to be susceptible to ≥1 study drug.

**Table 4:** All-cause mortality with C/T + metronidazole versus meropenem in patients with complex intra-abdominal infection.

Study (Year)	Outcome	C/T + Metronidazole (% [n/N])	Meropenem (% [n/N])	Percent Difference, % (95% CI)
**ASPECT-cIAI**				
**Solomkin (2015) [19]**	**cIAI (ITT) ^a^**			
	All-cause mortality	2.3 (11/482)	1.6 (8/497)	NR
**NCT01147640**				
**Lucasti (2014) [21]**	**cIAI (mITT) ^b^**			
	All-cause mortality	3.65 (3/82)	0	NR
**NCT03830333**				
**Sun (2022) [22]**	**cIAI (ITT) ^a^**			
	All-cause mortality	0 (0/0)	0.7 (1/134)	−0.7 (−4.1 to 2.1)

Abbreviations: CI: confidence interval; cIAI: complicated intra-abdominal infection; ITT: intention-to-treat; mITT: modified intent-to-treat; NR: not reported. ^a^ ITT was defined as all randomized participants, regardless of whether they received study drug. ^b^ The modified intent-to-treat (mITT) population was defined as all randomized patients who received at least one dose of the study drug.

**Table 5:** Rates of clinical cure with C/T versus meropenem for patients with ventilated hospital-acquired bacterial pneumonia/ventilator-associated bacterial pneumonia (ASPECT-NP).

Study (Year)	Outcome	C/T (% [n/N])	Meropenem (% [n/N])	Percent Difference, % (95% CI)
**Kollef (2019) [10]**	**vHABP/VABP (ITT) ^a^**			
	Clinical cure at TOC	54.4 (197/362)	53.3 (194/364)	1.1 (−6.2 to 8.3)
	**VABP (ITT) ^a^**			
	Clinical cure at TOC	55.9 (147/263)	57.0 (146/256)	−1.1 (−9.6 to 7.4)
	**vHABP (ITT) ^a^**			
	Clinical cure at TOC	50.5 (50/99)	44.4 (48/108)	6.1 (−7.4 to 19.3)
**Martin-Loeches (2019) [18]**	**vHABP/VABP (ME) ^b^**			
	Clinical cure: Overall	75.2 (85/113)	66.7 (78/117)	8.6 (−3.19 to 19.94)

Abbreviations: CI: confidence interval; C/T: ceftolozane/tazobactam; ITT: intention-to-treat; ME: microbiological evaluable; N: number; TOC: test-of-cure; VABP: ventilator-associated bacterial pneumonia; vHABP: ventilated hospital-associated bacterial pneumonia. ^a^ ITT was defined as all randomized participants, regardless of whether they received study drug. ^b^ Microbiologically evaluable (ME) population was defined as participants who received study drug, adhered to protocol requirements, and had ≥1 Gram-negative or streptococcal respiratory pathogen from baseline lower respiratory tract (LRT) cultures.

**Table 6:** Rates of clinical cure with C/T + metronidazole versus meropenem in patients with complex intra-abdominal infection (ASPECT-cIAI).

Study (Year)	Efficacy	C/T + Metronidazole (% [n/N])	Meropenem (% [n/N])	Percent Difference, % (95% CI)
**ASPECT-cIAI**				
**Solomkin (2015) [19]**	**cIAI (ITT) ^a^**			
	Clinical cure at TOC visit	83.6 (NR)	86.2 (NR)	−2.6 (−7.08 to 1.87)
**NCT01147640**				
**Lucasti. (2014) [21]**	**cIAI (CE) ^b^**			
	Clinical cure	91.4 (64/70)	94.3 (33/35)	−2.9 (−23.5 to 18.0)
**NCT03830333**				
**Sun (2022) [22]**	**cIAI (CE) ^b^**			
	Clinical response at the TOC visit	95.2 (NR)	93.1 (NR)	2.1 (−4.7 to 8.8)

Abbreviations: CE: clinical evaluable; CI: confidence interval; cIAI: complicated intra-abdominal infection; C/T: ceftolozane/tazobactam; NR: not reported; TOC: test-of-cure. ^a^ ITT was defined as all randomized participants, regardless of whether they received study drug. ^b^ Clinically evaluable (CE) population was defined as the participants who received study drug, adhered to the study protocol through the TOC visit, and had an evaluable clinical outcome at the TOC visit or clinical failure prior to the TOC visit.

**Table 7:** Clinical response rates of C/T versus levofloxacin in patients with complex urinary tract infection and pyelonephritis (ASPECT-cUTI).

Study (Year)	Outcome	C/T (% [n/N])	Levofloxacin (% [n/N])	Percent Difference, % (95% CI)
**Wagenlehner (2015) [23]**	**cUTI + Pyelonephritis (mMITT) ^a^**			
	Clinical cure (Overall population)	92.0 (366/398)	88.6 (356/402)	3.4 (−0.7 to 7.6)
	**cUTI + Pyelonephritis (Per-Protocol)**			
	Clinical cure (overall population)	95.9 (327/341)	93.2 (329/353)	2.7 (−0.8 to 6.2)
**Huntington (2016) [24]**	**cUTI + Pyelonephritis caused by levofloxacin-resistant pathogens (mMITT) ^a^**			
	Clinical response at the TOC visit (Overall population)	90.0 (90/100)	76.8 (86/112)	13.2 (3.1 to 22.9)
	Clinical response at the TOC visit (cUTI only)	82.8 (24/29)	68.6 (24/35)	14.2 (−7.4 to 33.3)
	Clinical response at the TOC visit (Pyelonephritis only)	93.0 (66/71)	80.5 (62/77)	12.4 (1.3 to 23.4)

Abbreviations: CI: confidence interval; cUTI: complicated urinary tract infection; mMITT: microbiological modified intention-to-treat population; TOC: test-of-cure. ^a^ The microbiological modified intention-to-treat population (mMITT) included all randomized patients who received the study drug and had growth of one or two uropathogens of at least 105 cfu/mL in a pretreatment baseline urine culture.

**Table 8:** Rates of microbiological eradication with C/T versus meropenem for ventilated hospital-acquired bacterial pneumonia/ventilator-associated bacterial pneumonia (ASPECT-NP).

Study (Year)	Outcome	C/T (% [n/N])	Meropenem (% [n/N])	Percent Difference, % (95% CI)
**Timsit (2021) [12]**	**vHABP (mITT) ^a^**			
	Microbiological eradication at TOC	78.2 (43/55)	62.0 (44/71)	16.2 (−0.1 to 30.8)
	**vHABP (ME) ^b^**			
	Microbiological eradication at TOC	71.4 (15/21)	64.0 (16/25)	7.4 (−19.1 to 31.9)

Abbreviations: CI: confidence interval; C/T: ceftolozane/tazobactam; ME: microbiological evaluable; mITT: microbiological intention-to-treat population; N: number; TOC: test-of-cure; vHABP: ventilated hospital-acquired bacterial pneumonia. ^a^ mITT was defined as the ITT participants who received ≥1 dose of study treatment and with ≥1 Gram-negative or streptococcal respiratory pathogen from baseline lower respiratory tract (LRT) cultures confirmed to be susceptible to ≥1 study drug. ^b^ Microbiologically evaluable (ME) population was defined as participants who received study drug, adhered to protocol requirements, had ≥1 Gram-negative or streptococcal respiratory pathogen from baseline lower respiratory tract (LRT) cultures.

**Table 9:** Microbiological eradication rates with C/T versus levofloxacin in patients with complex urinary tract infection and pyelonephritis (ASPECT-cUTI).

Study (Year)	Outcome	C/T (% [n/N])	Levofloxacin (% [n/N])	Percent Difference, % (95% CI)
**Wagenlehner (2015) [23]**	**cUTI + Pyelonephritis (mMITT) ^a^**			
	Microbiological eradication (Overall population)	80.4 (320/398)	72.1 (290/402)	8.3 (2.4 to 14.1)
	**cUTI + Pyelonephritis (Per-Protocol)**			
	Microbiological eradication (Overall population)	86.2 (294/341)	77.6 (274/353)	8.6 (2.9 to 14.3)
**Huntington (2016) [24]**	**cUTI + Pyelonephritis (mMITT) ^a^**			
	Microbiological response at the TOC visit (Overall population)	63.0 (63/100)	43.8 (49/112)	19.3 (5.8 to 31.7)
	Microbiological response at the TOC visit (cUTI only)	62.1 (18/29)	17.1 (6/35)	44.9 (21.1 to 62.6)
	Microbiological response at the TOC visit (Pyelonephritis only)	63.4 (45/71)	55.8 (43/77)	7.5 (−8.2 to 22.6)

Abbreviations: CI: confidence interval; cUTI: complicated urinary tract infection; mMITT: microbiological modified intention-to-treat population; TOC: test-of-cure. ^a^ The microbiological modified intention-to-treat population (mMITT) included all randomized patients who received the study drug and had growth of one or two uropathogens of at least 105 cfu/mL in a pretreatment baseline urine culture.

## Data Availability

The full datasets generated and analyzed to inform the conclusions drawn within this manuscript during the current study are available from the corresponding author upon reasonable request.

## Supplementary Materials

The following supporting information can be downloaded at: https://www.mdpi.com/article/10.3390/antibiotics15020190/s1. Table S1: PICOTS criteria for the SLR on vHABP/VABP; Table S2: PICOTS criteria for the SLR on cIAI and cUTI; Table S3: List of data sources; Table S4: cIAI/cUTI search strategy for EMBASE in Ovid, 1974 to 17 July 2024; search executed 18 July 2024; Table S5: cIAI/cUTI search strategy for PubMed, 1992 to 18 July 2024, search executed 18 July 2024; Table S6: cIAI/cUTI search strategy for Cochrane Database of Systematic Reviews; search executed 18 July 2024; Table S7: cIAI/cUTI search strategy for Cochrane Central Register of Controlled Trials; search executed 18 July 2024; Table S8: HABP/VABP search strategy for EMBASE in Ovid, 1974 to 2018; search executed 27 September 2018; Table S9: HABP/VABP search strategy for MEDLINE in Ovid, 1946 to 2018; search executed 27 September 2018; Table S10: Search strategy for Cochrane Database of Systematic Reviews; search executed 27 September 2018; Table S11: HABP/VABP search strategy for EMBASE in Ovid, 1974 to 18 July 2024; search executed 18 July 2024; Table S12: HABP/VABP search strategy for MEDLINE in Ovid, 1946 to 16 July 2024; search executed 18 July 2024; Table S13: HABP/VABP search strategy for Cochrane Database of Systematic Reviews; search executed 18 July 2024; Table S14: All-cause mortality with C/T versus meropenem in patient subgroups with ventilated hospital-acquired bacterial pneumonia/ventilator-associated bacterial pneumonia (ASPECT-NP); Table S15: Rates of clinical cure with C/T versus meropenem for patients subgroups with ventilated hospital-acquired bacterial pneumonia/ventilator-associated bacterial pneumonia (ASPECT-NP); Table S16: Rates of clinical cure with C/T + metronidazole versus meropenem in patient subgroups with complex intra-abdominal infection (ASPECT-cIAI); Table S17: Rates of microbiological eradication with C/T versus meropenem for patient subgroups with ventilated hospital-acquired bacterial pneumonia/ventilator-associated bacterial pneumonia (ASPECT-NP); Table S18: PRISMA Checklist.
