# Supplementary material for: Ceftolozane/Tazobactam for Complex and Resistant Infections: Systematic Reviews of Comparative Efficacy Studies
Source: Antibiotics (Basel). 2026 Feb 9;15(2):190. doi: 10.3390/antibiotics15020190 (PMC12937302; doi:10.3390/antibiotics15020190)
Supplement: Supplementary file 1 [file antibiotics-15-00190-s001.zip › antibiotics-4028367-supplementary.pdf]

Supplementary Material

Table S1. PICOTS criteria for the SLR on vHABP/VABP

PICOTS Components	Interpretation/Requirements
P - Population	• Patients aged ≥18 years<br>• Require hospitalization and treatment for a bacterial infection for HABP/VABP
I - Intervention	• Ceftolozane/Tazobactam
C - Comparator(s)	• Placebo<br>• Any antibiotic, unrestricted selection for comparators
O - Outcome(s)	Efficacy:<br>• Clinical cure or success<br>• Microbiological cure or eradication<br>• Composite cure<br>• Mortality<br>• Length of stay (LOS) or Intensive care unit LOS<br>• Mechanical ventilation days<br>• Readmission<br>Safety:<br>• Adverse events (overall, treatment emergent, discontinuations, incidence rates, total number of events)
T - Time period	• Original SLR: inception - September 26, 2018<br>• SLR update: September 27, 2018 - July 18, 2024
S - Study design	• Phase 2 or 3, RCTs and CCTs (including blinded and open label studies)
Language	• English

Abbreviations: *CCTs: Controlled Clinical Trials; LOS: Length of Stay; PICOTS: Population, Intervention, Comparison, Outcomes, Time period and Study design; RCTs: Randomized Controlled Trials; SLR: Systematic Literature Review; HABP: Hospital-Acquired Bacterial Pneumonia; VABP: Ventilator-Acquired Bacterial Pneumonia.*

Confidential - Not for Public Consumption or Distribution

Table S2. PICOTS criteria for the SLR on cIAI and cUTI

PICOTS Components	Interpretation/Requirements
P - Population	• Patients aged ≥18 years<br>• Require hospitalization and treatment for a bacterial infection for cIAI or cUTI
I - Intervention	• Ceftolozane/Tazobactam
C - Comparators	• Placebo<br>• Any antibiotic, unrestricted selection for comparators
O - Outcome(s)	Efficacy:<br>• Clinical cure or success<br>• Microbiological cure or eradication<br>• Composite cure<br>• Mortality<br>• Length of stay (LOS) or Intensive care unit LOS<br>• Mechanical ventilation days<br>• Readmission<br>Safety:<br>• Adverse events (overall, treatment emergent, discontinuations, incidence rates, total number of events)
T - Time	2021-2024
S - Study design	• Phase 2 or 3, RCTs and CCTs (including blinded and open label studies)
Language	• English

***Abbreviations**: CCTs; Controlled Clinical Trials; cIAI: Complicated Intra-Abdominal Infection; cUTI: Complicated Urinary Tract Infections; LOS: length of stay PICOTS: Population, Intervention, Comparison, Outcome, Time period and Study Design; RCT: Randomized Controlled Trial.*

Confidential - Not for Public Consumption or Distribution

Table S3. List of data sources

**Data Source**	**Sources of HABP/VABP Clinical SLR**	**Sources of cIAI/cUTI Clinical SLR**
Databases	MEDLINE(R) ALL (Ovid) (1946 – July 16, 2024)<br>EMBASE (Ovid) (1974 – July 18, 2024)<br>Cochrane Library (CENTRAL)	PubMed (1992 – July 18, 2024)<br>EMBASE (Ovid) (1974 – July 17, 2024)<br>Cochrane Library (CENTRAL and CDSR)
Conference proceedings (2018 to 2024)	European Society of Clinical Microbiology and Infectious Diseases (ECCMID)<br>Infectious Disease (ID) Week<br>American Society for Microbiology (ASM)<br>Society of Critical Care Medicine (SCCM)<br>American Thoracic Society (ATS)	European Society of Clinical Microbiology and Infectious Diseases (ECCMID)<br>ID Week<br>American Society for Microbiology (ASM) Microbe
Gray literature (Others)	Bibliographic searching of relevant SLRs	Bibliographic searching of relevant SLRs
Clinical trial Searching	clinicaltrials.gov website	clinicaltrials.gov website

Abbreviations: ASM: American Society for Microbiology; ATS: American Thoracic Society; CDSR: Cochrane Database of Systematic Reviews; cIAI: Complicated Intra-Abdominal Infection; cUTI: Complicated Urinary Tract Infections; ECCMID: European Society of Clinical Microbiology and Infectious Diseases; HABP: Ventilated Hospital-Acquired Bacterial Pneumonia; ID: Infectious Disease; SLR: Systematic Literature Review; VABP: Ventilator-Acquired Bacterial Pneumonia.

Confidential - Not for Public Consumption or Distribution

Table S4. cIAI/cUTI Search strategy for EMBASE in Ovid 1974 to July 17, 2024, search executed July 18, 2024

No.	Criteria	Strings	Hits
1	Population	exp urinary tract infection/	148,164
2	Population	exp pyelonephritis/	25,701
3	Population	catheter infection/ or catheter associated urinary tract infection/	22,659
4	Population	(urinary tract infection$ or uti or utis).tw.	85,175
5	Population	(pyelonephr$ or bacteriuria$).tw.	24,655
6	Population	(catheter$ adj2 infection$).tw.	7,924
7	Population	((genitourin$ or ureter$ or ureth$ or urin$ or urolog$ or urogen$ or catheter) adj5 (infect$ or bacteria$ or microbiol$ or abscess$)).tw.	120,195
8	Population	(urin$ adj5 (abnormal$ or obstruct$ or fistula$ or calcul$ or stricture$)).tw.	31,122
9	Population	or/1-8	254,447
10	Population	exp abdominal Infection/	37,105
11	Population	exp appendicitis/	31,660
12	Population	exp diverticulitis/	10,604
13	Population	exp peritonitis/	65,560
14	Population	(intra abdominal infection$ or intra-abdominal infection$ or intraabdominal infection$).tw.	4,874
15	Population	((intraabdominal or intra-abdominal or abdominal) adj2 (infection$ or abscess$)).tw.	14,958
16	Population	appendicitis.tw.	26,281
17	Population	diverticulitis.tw.	10,989
18	Population	peritonitis.tw.	46,185
19	Population	or/10-18	143,812
20	Population	9 or 19	386,484
21	Intervention	ceftolozane plus tazobactam/	1,965
22	Intervention	(zerbaxa$ or cxa 201 or cxa201 or mk 7625a or mk7625a).ti,ab,kf,tn,dy.	113
23	Intervention	(ceftolozane adj5 tazobactam).ti,ab,kf,dy.	2,161
24	Intervention	or/21-23	2,178
25	Intervention	20 and 24	633
26	Study design	editorial/ or letter/ or note/	2,954,084
27	Study design	case report/	3,018,447
28	Study design	(rat or rats or mouse or mice or swine or porcine or murine or sheep or lambs or pigs or piglets or rabbit or rabbits or cat or cats or dog or dogs or cattle or bovine or monkey or monkeys or trout or marmoset$).ti. and animal experiment/	1,259,695
29	Study design	'animal experiment'/ not ('human experiment'/ or	2,649,600

Confidential - Not for Public Consumption or Distribution

No.	Criteria	Strings	Hits
		'human'/)	
30	Study design	or/26-29	8,356,230
31	Combined	25 not 30	**543**

Confidential - Not for Public Consumption or Distribution

Table S5. cIAI/cUTI Search strategy for PubMed 1992 to July 18, 2024, search executed July 18, 2024

No	Criteria	Strings	Hits
1	Population	"Urinary Tract Infections"[MeSH Terms]	51,972
2	Population	"pyelonephritis"[MeSH Terms]	15,186
3	Population	"Catheter-Related Infections"[MeSH Terms:noexp]	6,522
4	Population	"Intraabdominal Infections"[MeSH Terms]	51,005
5	Population	"abdominal abscess"[MeSH Terms]	12,977
6	Population	("urinary tract infection*"[Title/Abstract] OR "uti"[Title/Abstract] OR "utis"[Title/Abstract]) OR ("pyelonephr*"[Title/Abstract] OR "bacteriuria*"[Title/Abstract]) OR ("catheter*"[Title/Abstract] AND "infection*"[Title/Abstract]) OR (("genitourin*"[Title/Abstract] OR "ureter*"[Title/Abstract] OR "ureth*"[Title/Abstract] OR "urin*"[Title/Abstract] OR "urolog*"[Title/Abstract] OR "urogen*"[Title/Abstract] OR "catheter"[Title/Abstract]) AND ("infect*"[Title/Abstract] OR "bacteria*"[Title/Abstract] OR "microbiol*"[Title/Abstract] OR "abscess*"[Title/Abstract])) OR ("urin*"[Title/Abstract] AND ("abnormal*"[Title/Abstract] OR "obstruct*"[Title/Abstract] OR "fistula*"[Title/Abstract] OR "calcul*"[Title/Abstract] OR "stricture*"[Title/Abstract])) OR ("intra abdominal infection*"[Title/Abstract] OR "intra abdominal infection*"[Title/Abstract] OR "intraabdominal infection*"[Title/Abstract]) OR (("intraabdominal"[Title/Abstract] OR "intra-abdominal"[Title/Abstract] OR "abdominal"[Title/Abstract]) AND ("infection*"[Title/Abstract] OR "abscess*"[Title/Abstract])) OR ("appendicitis"[Title/Abstract] OR "diverticulitis"[Title/Abstract] OR "peritonitis"[Title/Abstract])	320,638
7	Population	#1 OR #2 OR #3 OR #4 OR #5 OR #6	361,566
8	Intervention	"zerbaxa*"[Title/Abstract] OR "cxa 201"[Title/Abstract] OR "cxa201"[Title/Abstract] OR "mk 7625a"[Title/Abstract] OR "mk7625a"[Title/Abstract] OR "ceftolozane tazobactam"[Title/Abstract:~4] OR ("penicillanic acid"[MeSH Terms] AND	3,151

Confidential - Not for Public Consumption or Distribution

No	Criteria	Strings	Hits
		"tazobactam"[MeSH Terms])	
9	Intervention	#7 AND #8	606
10	Study design	"editorial"[Publication Type] OR "letter"[Publication Type] OR "case report"[Title]	2,285,977
11	Study design	animals[mesh] NOT humans[mesh]	5,243,358
12	Combined	#9 NOT (#10 OR #11)	**544**

Confidential - Not for Public Consumption or Distribution

Table S6. cIAI/cUTI Search strategy for Cochrane Database of Systematic Reviews, search executed July 18, 2024

No.	Criteria	Strings	Hits
1	Population	appendicitis:ti,ab OR diverticulitis:ti,ab OR peritonitis:ti,ab	3,819
2	Population	((intraabdominal:ti,ab OR "intra abdominal":ti,ab OR abdominal:ti,ab) AND (infection*:ti,ab OR abscess*:ti,ab))	5,476
3	Population	(("intra abdominal" NEXT infection*):ti,ab OR ("intraabdominal" NEXT infection*):ti,ab)	580
4	Population	[mh "Intraabdominal Infections"] OR [mh "abdominal abscess"]	1,814
5	Population	#1 OR #2 OR #3 OR #4	9,052
6	Population	(urin*:ti,ab AND (abnormal*:ti,ab OR obstruct*:ti,ab OR fistula*:ti,ab OR calcul*:ti,ab OR stricture*:ti,ab))	1,1079
7	Population	((genitourin*:ti,ab OR ureter*:ti,ab OR ureth*:ti,ab OR urin*:ti,ab OR urolog*:ti,ab OR urogen*:ti,ab OR catheter:ti,ab) AND (infect*:ti,ab OR bacteria*:ti,ab OR microbiol*:ti,ab OR abscess*:ti,ab))	1,7370
8	Population	(catheter*:ti,ab AND infection*:ti,ab)	4,858
9	Population	(pyelonephr*:ti,ab OR bacteriuria*:ti,ab)	1,729
10	Population	(("urinary tract" NEXT infection*):ti,ab OR uti:ti,ab OR utis:ti,ab)	7,594
11	Population	[mh "Urinary Tract Infections"] OR [mh Pyelonephritis] OR [mh ^"Catheter-Related Infections"]	3,941
12	Population	#6 OR #7 OR #8 OR #9 OR 10 OR #11	28,089
13	Population	#5 OR #12	35,710
14	Intervention	MeSH descriptor: [Penicillanic Acid] this term only	339
15	Intervention	MeSH descriptor: [Tazobactam] this term only	215
16	Intervention	#14 AND #15	146
17	Intervention	(ceftolozane NEAR/4 tazobactam):ti,ab	73
18	Intervention	(zerbaxa* OR "cxa 201" OR cxa201 OR "mk 7625a" OR mk7625a):ti,ab	19
19	Intervention	#16 OR #17 OR #18	210
20	Combined	#13 AND #19 in Cochrane Reviews	**0**

Table S7. cIAI/cUTI Search strategy for Cochrane Central Register of Controlled Trials, search executed July 18, 2024

No.	Criteria	Strings	Hits
1	Population	appendicitis:ti,ab OR diverticulitis:ti,ab OR peritonitis:ti,ab	3,845
2	Population	((intraabdominal:ti,ab OR "intra abdominal":ti,ab OR abdominal:ti,ab) AND (infection*:ti,ab OR	5,558

Confidential - Not for Public Consumption or Distribution

No.	Criteria	Strings	Hits
		abscess*:ti,ab))	
3	Population	(("intra abdominal" NEXT infection*):ti,ab OR ("intraabdominal" NEXT infection*):ti,ab)	583
4	Population	[mh "Intraabdominal Infections"] OR [mh "abdominal abscess"]	1,817
5	Population	OR #1 OR #2 OR #3 OR #4	9,154
6	Population	(urin*:ti,ab AND (abnormal*:ti,ab OR obstruct*:ti,ab OR fistula*:ti,ab OR calcul*:ti,ab OR stricture*:ti,ab))	11324
7	Population	((genitourin*:ti,ab OR ureter*:ti,ab OR ureth*:ti,ab OR urin*:ti,ab OR urolog*:ti,ab OR urogen*:ti,ab OR catheter:ti,ab) AND (infect*:ti,ab OR bacteria*:ti,ab OR microbiol*:ti,ab OR abscess*:ti,ab))	17,624
8	Population	(catheter*:ti,ab AND infection*:ti,ab)	4,934
9	Population	(pyelonephr*:ti,ab OR bacteriuria*:ti,ab)	1,741
10	Population	(("urinary tract" NEXT infection*):ti,ab OR uti:ti,ab OR utis:ti,ab)	7,678
11	Population	[mh "Urinary Tract Infections"] OR [mh Pyelonephritis] OR [mh ^"Catheter-Related Infections"]	3,951
12	Population	#6 OR #7 or #8 OR #9 OR #10 OR #11	28,563
13	Population	#5 OR #12	36,264
14	Intervention	MeSH descriptor: [Penicillanic Acid] this term only	340
15	Intervention	MeSH descriptor: [Tazobactam] this term only	216
16	Intervention	#14 AND #15	146
17	Intervention	(ceftolozane NEAR/4 tazobactam):ti,ab	74
18	Intervention	(zerbaxa* OR "cxa 201" OR cxa201 OR "mk 7625a" OR mk7625a):ti,ab	19
19	Intervention	#16 OR #17 OR #18	211
20	Combined	#13 AND #19 in Trials	**86**

Table S8. HABP/VABP Search strategy for EMBASE in Ovid 1974 to 2018, search executed September 27, 2018

No.	Criteria	Strings	Hits
1	Study design	Clinical Trial/	949889
2	Study design	Randomized Controlled Trial/	513634
3	Study design	controlled clinical trial/	457965
4	Study design	multicenter study/	194205
5	Study design	Phase 3 clinical trial/	35674
6	Study design	Phase 4 clinical trial/	3083
7	Study design	exp RANDOMIZATION/	79618
8	Study design	Single Blind Procedure/	32399
9	Study design	Double Blind Procedure/	152931
10	Study design	Crossover Procedure/	56521
11	Study design	PLACEBO/	323113

Confidential - Not for Public Consumption or Distribution

No.	Criteria	Strings	Hits
12	Study design	randomi?ed controlled trial$.tw.	185951
13	Study design	rct.tw.	29424
14	Study design	(random$ adj2 allocat$).tw.	37528
15	Study design	single blind$.tw.	21569
16	Study design	double blind$.tw.	190283
17	Study design	((treble or triple) adj blind$).tw.	835
18	Study design	placebo$.tw.	277381
19	Study design	Prospective Study/	469836
20	Study design	or/1-19	2017320
21	Study design	Case Study/	56327
22	Study design	case report.tw.	362426
23	Study design	abstract report/ or letter/	1070232
24	Study design	Conference proceeding.pt.	0
25	Study design	Conference abstract.pt.	3156716
26	Study design	Editorial.pt.	576421
27	Study design	Letter.pt.	1031422
28	Study design	Note.pt.	723321
29	Study design	or/21-28	5894245
30	Study design	20 not 29	1542748
31	Population	exp pneumonia, bacterial/	27430
32	Population	(bacterial AND pneumonia).mp.	44889
33	Population	31 or 32	56297
34	Population	((hospital OR ventilator OR nosocomial) AND pneumonia).mp.	70710
35	Population	33 AND 34	14517
36	Population	exp pneumonia, ventilator-associated/	9046
37	Population	35 or 36	21466
38	Intervention	(meropenem or Merrem or Penem or Ronem or SM 7338 or SM-7338).mp.	28666
39	Intervention	(ceftolozane or 'CXA-201' or 'cb-500,201' OR cb500201 OR 'cxa-201' or cxa201 or 'cxa 101 plus tazobactam' or 'ceftolozane tazobactam' or 'ceftolozane plus tazobactam' or 'ceftolozane-tazobactam' or (ceftolozane adj2 tazobactam)).mp.	520
40	Intervention	piperacillin plus tazobactam/	22478
41	Intervention	('piperacillin plus tazobactam' or (Piperacillin AND tazobactam) or (piperacillin adj2 tazobactam) or 'piperacillin-tazobactam' or Tazocin or Zosyn or tazopril or tazobac or tazonam or tazocillin or tazocilline or tazomax or tazocel).mp.	25888
42	Intervention	(cefepim or cefepime or cefepime hyrdrochloride or Quadrocef or Maxipime or Axepim or BMY 28142 or BMY-28142).ti,ab,kw.	4970
43	Intervention	exp levofloxacin/	31444
44	Intervention	(levofloxacin or Levaquin or Tavanic or iquix or mosardal or nofaxin or levox or 'cravit aphtalmic' or levokacin or lesacin or tavanic or quixin or cravit or	32476

Confidential - Not for Public Consumption or Distribution

No.	Criteria	Strings	Hits
		reskuin or levaquin or floxel or leroxacin or volequin or elequine).mp.	
45	Intervention	exp ceftazidime/	37466
46	Intervention	(ceftazidime OR Fortum or LY-139381 OR LY 139381 OR LY139381 OR tazidime OR Fortaz OR GR-20263 OR GR 20263 OR GR20263).mp.	38869
47	Intervention	exp imipenem/	34173
48	Intervention	(imipenem OR Primaxin or MK-0787 OR MK 0787 OR MK0787 or N-Formimidoylthienamycin OR N Formimidoylthienamycin).mp.	38933
49	Intervention	ciprofloxacin/	89167
50	Intervention	(ciprofloxacin or Ciloxan or Cipro or Neofloxin).mp.	92385
51	Intervention	exp prulifloxacin/	472
52	Intervention	(prulifloxacin or Quisnon or Unidrox or Prixina or Glimbax).mp.	483
53	Intervention	exp ertapenem/	6093
54	Intervention	(ertapenem or Invanz).mp.	6226
55	Intervention	exp doripenem/	2341
56	Intervention	(doripenem or Doribax or Finibax).mp.	2388
57	Intervention	exp ceftriaxone/	52857
58	Intervention	(ceftriaxone or Rocephin or Epicephin).mp.	54334
59	Intervention	exp amikacin/	42084
60	Intervention	(amikacin OR Yectamid OR Amikacina OR Amikafur OR amikalem or Amikason's OR Amikin OR Biklin OR Biclin OR Amiklin OR Amukin OR BB-K8 or BB K8 OR BBK8 OR BB-K 8 OR BB K 8 OR BBK 8 OR gamikal OR kanbine OR oprad).mp	43495
61	Intervention	exp gentamicins/	97970
62	Intervention	(gentamycin OR garamycin OR gentacyclo OR Gentavet OR genticin OR G-Myticin OR Gmyticin OR G Myticin OR Gentamicin OR Gentamycin).mp.	102779
63	Intervention	exp tobramycin/	30439
64	Intervention	(tobramycin OR obracin OR tobracin OR brulamycin OR nebcin OR nebicin OR nebramycin).mp.	32208
65	Intervention	exp plazomicin/	176
66	Intervention	(plazomicin or Zemdri).mp.	183
67	Intervention	('colistin plus polymyxin' or 'colistin-polymyxin' or (colistin adj2 polymyxin)).mp.	370
68	Intervention	exp colistin/	14998
69	Intervention	("Colisticin" or "Polymyxin E" or "Colimycin" or "Coly-Mycin" or "Totazina" or "Colistin Sulfate" or "Sulfate, Colistin").mp.	749
70	Intervention	exp polymyxins/	5715
71	Intervention	(“Polymyxin” or “Polymyxin M”).mp.	16738
72	Intervention	exp cefiderocol/	47
73	Intervention	or/38-72	286844
74	Combined	30 and 37 and 73	1141
75	Combined	limit 74 to yr="2000-Current"	1045

Confidential - Not for Public Consumption or Distribution

Confidential - Not for Public Consumption or Distribution

Table S9. HABP/VABP Search strategy for MEDLINE in Ovid 1946 to 2018, search executed September 27, 2018

No.	Criteria	Strings	Hits
1	Study design	Randomized Controlled Trials as Topic/	118569
2	Study design	randomized controlled trial/	468737
3	Study design	Random Allocation/	95976
4	Study design	Double Blind Method/	147619
5	Study design	Single Blind Method/	25713
6	Study design	clinical trial/	512486
7	Study design	clinical trial, phase i.pt	18390
8	Study design	clinical trial, phase ii.pt	29645
9	Study design	clinical trial, phase iii.pt	14183
10	Study design	clinical trial, phase iv.pt	1594
11	Study design	controlled clinical trial.pt	92661
12	Study design	randomized controlled trial.pt	468737
13	Study design	multicenter study.pt	239474
14	Study design	clinical trial.pt	512486
15	Study design	exp Clinical Trials as topic/	317783
16	Study design	or/1-15	1256543
17	Study design	(clinical adj trial$).tw	315644
18	Study design	((singl$ or doubl$ or treb$ or tripl$) adj (blind$3 or mask$3)).tw	159000
19	Study design	PLACEBOS/	34073
20	Study design	placebo$.tw	198184
21	Study design	randomly allocated.tw	24902
22	Study design	(allocated adj2 random$).tw	27978
23	Study design	or/17-22	565124
24	Study design	16 or 23	1484868
25	Study design	case report.tw	276679
26	Study design	letter/	1000335
27	Study design	historical article/	347230
28	Study design	or/25-27	1609841
29	Study design	24 not 28	1451138
30	Population	exp pneumonia, bacterial/	20520
31	Population	(bacterial AND pneumonia).mp.	36325
32	Population	30 or 31	41897
33	Population	((hospital OR ventilator OR nosocomial) AND pneumonia).mp.	29441
34	Population	32 AND 33	9860
35	Population	exp pneumonia, ventilator-associated/	2999
36	Population	34 or 35	11617
37	Intervention	(meropenem or Merrem or Penem or Ronem or SM 7338 or SM-7338).mp.	6220
38	Intervention	(ceftolozane or 'CXA-201' or 'cb-500,201' OR cb500201 OR 'cxa-201' or cxa201 or 'cxa 101 plus tazobactam' or 'ceftolozane tazobactam' or 'ceftolozane plus tazobactam' or 'ceftolozane-tazobactam' or (ceftolozane adj2 tazobactam)).mp.	277

Confidential - Not for Public Consumption or Distribution

No.	Criteria	Strings	Hits
39	Intervention	('piperacillin plus tazobactam' or (Piperacillin AND tazobactam) or (piperacillin adj2 tazobactam) or 'piperacillin-tazobactam' or Tazocin or Zosyn or tazopril or tazobac or tazonam or tazocillin or tazocilline or tazomax or tazocel).mp.	3811
40	Intervention	(cefepim or cefepime or cefepime hyrdrochloride or Quadrocef or Maxipime or Axepim or BMY 28142 or BMY-28142).ti,ab,kw.	3020
41	Intervention	exp levofloxacin/	2982
42	Intervention	(levofloxacin or Levaquin or Tavanic or iquix or mosardal or nofaxin or levox or 'cravit aphtalmic' or levokacin or lesacin or tavanic or quixin or cravit or reskuin or levaquin or floxel or leroxacin or volequin or elequine).mp.	6935
43	Intervention	exp ceftazidime/	3648
44	Intervention	(ceftazidime OR Fortum or LY-139381 OR LY 139381 OR LY139381 OR tazidime OR Fortaz OR GR-20263 OR GR 20263 OR GR20263).mp.	8896
45	Intervention	exp imipenem/	3867
46	Intervention	(imipenem OR Primaxin or MK-0787 OR MK 0787 OR MK0787 or N-Formimidoylthienamycin OR N Formimidoylthienamycin).mp.	10224
47	Intervention	ciprofloxacin/	12232
48	Intervention	(ciprofloxacin or Ciloxan or Cipro or Neofloxin).mp.	25826
49	Intervention	(prulifloxacin or Quisnon or Unidrox or Prixina or Glimbax).mp.	142
50	Intervention	(ertapenem or Invanz).mp.	1369
51	Intervention	(doripenem or Doribax or Finibax).mp.	600
52	Intervention	exp ceftriaxone/	5517
53	Intervention	(ceftriaxone or Rocephin or Epicephin).mp.	11330
54	Intervention	exp amikacin/	3924
55	Intervention	(amikacin OR Yectamid OR Amikacina OR Amikafur OR amikalem or Amikason's OR Amikin OR Biklin OR Biclin OR Amiklin OR Amukin OR BB-K8 or BB K8 OR BBK8 OR BB-K 8 OR BB K 8 OR BBK 8 OR gamikal OR kanbine OR oprad).mp	9488
56	Intervention	exp gentamicins/	18372
57	Intervention	(gentamycin OR garamycin OR gentacyclo OR Gentavet OR genticin OR G-Myticin OR Gmyticin OR G Myticin OR Gentamicin OR Gentamycin).mp.	25467
58	Intervention	exp tobramycin/	4095
59	Intervention	(tobramycin OR obracin OR tobracin OR brulamycin OR nebcin OR nebicin OR nebramycin).mp.	7509
60	Intervention	(plazomicin or Zemdri).mp.	57
61	Intervention	('colistin plus polymyxin' or 'colistin-polymyxin' or (colistin adj2 polymyxin)).mp.	310
62	Intervention	exp colistin/	3582

Confidential - Not for Public Consumption or Distribution

No.	Criteria	Strings	Hits
63	Intervention	("Colisticin" or "Polymyxin E" or "Colimycin" or "Coly-Mycin" or "Totazina" or "Colistin Sulfate" or "Sulfate, Colistin").mp.	711
64	Intervention	exp polymyxins/	8571
65	Intervention	(“Polymyxin” or “Polymyxin M”).mp.	7330
66	Intervention	cefiderocol.mp.	28
67	Intervention	or/37-66	99993
68	Combined	29 and 36 and 67	378
69	Combined	limit 68 to yr="2000-Current"	306

Confidential - Not for Public Consumption or Distribution

Table S10: Search strategy for Cochrane Database of Systematic Reviews, search executed September 27, 2018

No.	Criteria	Strings	Hits
1	Population	exp pneumonia, bacterial/	710
2	Population	(bacterial AND pneumonia).mp.	2153
3	Population	1 or 2	2249
4	Population	((hospital OR ventilator OR nosocomial) AND pneumonia).mp.	3561
5	Population	3 AND 4	853
6	Population	exp pneumonia, ventilator-associated/	319
7	Population	5 or 6	1041
8	Intervention	(meropenem or Merrem or Penem or Ronem or SM 7338 or SM-7338).mp.	518
9	Intervention	(ceftolozane or 'CXA-201' or 'cb-500,201' OR cb500201 OR 'cxa-201' or cxa201 or 'cxa 101 plus tazobactam' or 'ceftolozane tazobactam' or 'ceftolozane plus tazobactam' or 'ceftolozane-tazobactam' or (ceftolozane adj2 tazobactam)).mp.	31
10	Intervention	('piperacillin plus tazobactam' or (Piperacillin AND tazobactam) or (piperacillin adj2 tazobactam) or 'piperacillin-tazobactam' or Tazocin or Zosyn or tazopril or tazobac or tazonam or tazocillin or tazocilline or tazomax or tazocel).mp.	498
11	Intervention	(cefepim or cefepime or cefepime hyrdrochloride or Quadrocef or Maxipime or Axepim or BMY 28142 or BMY-28142).ti,ab,kw.	262
12	Intervention	exp levofloxacin/	524
13	Intervention	(levofloxacin or Levaquin or Tavanic or iquix or mosardal or nofaxin or levox or 'cravit aphtalmic' or levokacin or lesacin or tavanic or quixin or cravit or reskuin or levaquin or floxel or leroxacin or volequin or elequine).mp.	1284
14	Intervention	exp ceftazidime/	446
15	Intervention	(ceftazidime OR Fortum or LY-139381 OR LY 139381 OR LY139381 OR tazidime OR Fortaz OR GR-20263 OR GR 20263 OR GR20263).mp.	994
16	Intervention	exp imipenem/	282
17	Intervention	(imipenem OR Primaxin or MK-0787 OR MK 0787 OR MK0787 or N-Formimidoylthienamycin OR N Formimidoylthienamycin).mp.	630
18	Intervention	ciprofloxacin/	1057
19	Intervention	(ciprofloxacin or Ciloxan or Cipro or Neofloxin).mp.	2376
20	Intervention	(prulifloxacin or Quisnon or Unidrox or Prixina or Glimbax).mp.	46
21	Intervention	(ertapenem or Invanz).mp.	161
22	Intervention	(doripenem or Doribax or Finibax).mp.	69
23	Intervention	exp ceftriaxone/	662
24	Intervention	(ceftriaxone or Rocephin or Epicephin).mp.	1426
25	Intervention	exp amikacin/	349

Confidential - Not for Public Consumption or Distribution

No.	Criteria	Strings	Hits
26	Intervention	(amikacin OR Yectamid OR Amikacina OR Amikafur OR amikalem or Amikason's OR Amikin OR Biklin OR Biclin OR Amiklin OR Amukin OR BB-K8 or BB K8 OR BBK8 OR BB-K 8 OR BB K 8 OR BBK 8 OR gamikal OR kanbine OR oprad).mp	868
27	Intervention	exp gentamicins/	1143
28	Intervention	(gentamycin OR garamycin OR gentacyclo OR Gentavet OR genticin OR G-Myticin OR Gmyticin OR G Myticin OR Gentamicin OR Gentamycin).mp.	1904
29	Intervention	exp tobramycin/	567
30	Intervention	(tobramycin OR obracin OR tobracin OR brulamycin OR nebcin OR nebicin OR nebramycin).mp.	1331
31	Intervention	(plazomicin or Zemdri).mp.	6
32	Intervention	('colistin plus polymyxin' or 'colistin-polymyxin' or (colistin adj2 polymyxin)).mp.	5
33	Intervention	exp colistin/	148
34	Intervention	("Colisticin" or "Polymyxin E" or "Colimycin" or "Coly-Mycin" or "Totazina" or "Colistin Sulfate" or "Sulfate, Colistin").mp.	60
35	Intervention	exp polymyxins/	366
36	Intervention	(“Polymyxin” or “Polymyxin M”).mp.	395
37	Intervention	cefiderocol.mp.	4
38	Intervention	or/8-37	94639849
39	Combined	7 and 38	288
40	Combined	limit 39 to yr="2000-Current"	231

The search strategies of the updated SLR were executed on July 18, 2024.

Table S11. HABP/VABP Search strategy for EMBASE in Ovid 1974 to July 18, 2024, search executed July 18, 2024

No.	Criteria	Strings	Hits
1	Study design	clinical Trial/	1,085,368
2	Study design	randomized controlled trial/	831,328
3	Study design	controlled clinical trial/	473,529
4	Study design	multicenter study/	396,913
5	Study design	Phase 3 clinical trial/	76,577
6	Study design	Phase 4 clinical trial/	7,454
7	Study design	exp Randomization/	99,937
8	Study design	single blind procedure/	55,480
9	Study design	double blind procedure/	221,104
10	Study design	crossover procedure/	78,762
11	Study design	placebo/	415,109
12	Study design	Randomi?ed controlled trial$.tw.	351,326
13	Study design	rct.tw.	58,523
14	Study design	(random$ adj2 allocat$).tw.	57,977
15	Study design	Single blind$.tw.	33,491
16	Study design	Double blind$.tw.	253,676
17	Study design	((treble or triple) adj blind$).tw.	2,163

Confidential - Not for Public Consumption or Distribution

No.	Criteria	Strings	Hits
18	Study design	placebo$.tw.	382,062
19	Study design	Prospective study/	926,971
20	Study design	or/1-19	3,080,290
21	Study design	case study/	101,854
22	Study design	case report.tw.	576,530
23	Study design	abstract report/ or letter/	1,336,925
24	Study design	conference proceeding.pt.	0
25	Study design	conference abstract.pt.	5,200,097
26	Study design	editorial.pt.	812,398
27	Study design	letter.pt.	1,330,146
28	Study design	note.pt.	992,883
29	Study design	or/21-28	8,915,317
30	Study design	20 not 29	2,197,583
31	Population	exp pneumonia, bacterial/	38,454
32	Population	(bacterial and pneumonia).mp.	66,606
33	Population	31 or 32	82,136
34	Population	((hospital or ventilator or nosocomial) and pneumonia).mp.	130,011
35	Population	33 and 34	24,214
36	Population	exp pneumonia, ventilator-associated/	14,762
37	Population	35 or 36	35,428
38	Intervention	meropenem/	53,603
39	Intervention	(meropenem or Merrem or Penem or Ronem or SM 7338 or SM- 7338).mp.	56,483
40	Intervention	ceftolozane/	457
41	Intervention	'ceftolozane plus tazobactam'/	1,965
42	Intervention	(ceftolozane or 'CXA-201' or 'cb-500,201' or cb500201 or 'cxa- 201' or cxa201 or 'cxa 101 plus tazobactam' or 'ceftolozane tazobactam' or 'ceftolozane plus tazobactam' or 'ceftolozane-tazobactam' or (ceftolozane adj2 tazobactam)).mp.	2,376
43	Intervention	'piperacillin plus tazobactam'/	42,950
44	Intervention	('piperacillin plus tazobactam' or (Piperacillin and tazobactam) or (piperacillin adj2 tazobactam) or 'piperacillin-tazobactam' or Tazocin or Zosyn or tazopril or tazobac or tazonam or tazocillin or tazocilline or tazomax or tazocel).mp.	47,713
45	Intervention	cefepime/	31,625
46	Intervention	(cefepim or cefepime or cefepime hyrdrochloride or Quadrocef or Maxipime or Axepim or BMY 28142 or BMY-28142).ti,ab,kw.	8,538
47	Intervention	exp levofloxacin/	53,334
48	Intervention	(levofloxacin or Levaquin or Tavanic or iquix or mosardal or nofaxin or levox or 'cravit aphtalmic' or levokacin or lesacin or tavanic or quixin or cravit or reskuin or levaquin or floxel or leroxacin or volequin or elequine).mp.	54,810
49	Intervention	exp ceftazidime/	54,517

Confidential - Not for Public Consumption or Distribution

No.	Criteria	Strings	Hits
50	Intervention	(ceftazidime or Fortum or LY-139381 or LY 139381 or LY139381 or tazidime or Fortaz or GR-20263 or GR 20263 or GR20263).mp.	58,155
51	Intervention	exp imipenem/	50,469
52	Intervention	(imipenem or Primaxin or MK-0787 or "MK 0787" or MK0787 or N-Formimidoylthienamycin or N Formimidoylthienamycin).mp.	57,028
53	Intervention	ciprofloxacin/	128,719
54	Intervention	(ciprofloxacin or Ciloxan or Cipro or Neofloxin).mp.	133,506
55	Intervention	exp prulifloxacin/	545
56	Intervention	(prulifloxacin or Quisnon or Unidrox or Prixina or Glimbax).mp.	559
57	Intervention	exp ertapenem/	11,765
58	Intervention	(ertapenem or Invanz).mp.	12,017
59	Intervention	exp doripenem/	3,683
60	Intervention	(doripenem or Doribax or Finibax).mp.	3,758
61	Intervention	exp ceftriaxone/	85,186
62	Intervention	(ceftriaxone or Rocephin or Epicephin).mp.	87,391
63	Intervention	exp amikacin/	61,521
64	Intervention	(amikacin or Yectamid or Amikacina or Amikafur or amikalem or Amikason's or Amikin or Biklin or Biclin or Amiklin or Amukin or BB-K8 or BB K8 or BBK8 or BB-K 8 or BB K 8 or BBK 8 or gamikal or kanbine or oprad).mp.	63,453
65	Intervention	exp gentamicins/	133,403
66	Intervention	(gentamycin or garamycin or gentacyclo or Gentavet or genticin or G-Myticin or Gmyticin or G Myticin or Gentamicin or Gentamycin).mp.	139,483
67	Intervention	exp tobramycin/	39,200
68	Intervention	(tobramycin or obracin or tobracin or brulamycin or nebcin or nebicin or nebramycin).mp.	41,788
69	Intervention	exp plazomicin/	569
70	Intervention	(plazomicin or Zemdri).mp.	600
71	Intervention	('colistin plus polymyxin' or 'colistin-polymyxin' or (colistin adj2 polymyxin)).mp.	677
72	Intervention	exp colistin/	27,004
73	Intervention	("Colisticin" or "Polymyxin E" or "Colimycin" or "Coly-Mycin" or "Totazina" or "Colistin Sulfate" or "Sulfate, Colistin").mp.	1,072
74	Intervention	exp polymyxins/	7,771
75	Intervention	("Polymyxin" or "Polymyxin M").mp.	24,174
76	Intervention	exp cefiderocol/	1,363
77	Intervention	or/38-76	422,783
78	Combined	30 and 37 and 77	1,676
79	Time period	(2018$ or 2019$ or 2020$ or 2021$ or 2022$ or 2023$ or 2024$).em.	14,868,310
80	Time period	("2018" or "2019" or "2020" or "2021" or "2022" or "2023" or "2024").yr.	11,714,187
81	Combined	78 and (79 or 80)	703

Confidential - Not for Public Consumption or Distribution

No.	Criteria	Strings	Hits
82	Language	limit 81 to english language	692

Confidential - Not for Public Consumption or Distribution

Table S12. HABP/VABP Search strategy for MEDLINE in Ovid 1946 to July 16, 2024, search executed July 18, 2024

No.	Criteria	Strings	Hits
1	Study design	Randomized Controlled Trials as Topic/	171,844
2	Study design	randomized controlled trial/	617,667
3	Study design	random allocation/	107,412
4	Study design	Double Blind Method/	179,545
5	Study design	Single Blind Method/	33,738
6	Study design	clinical trial/	540,236
7	Study design	clinical trial, phase i.pt.	26,189
8	Study design	clinical trial, phase ii.pt.	41,657
9	Study design	clinical trial, phase iii.pt.	23,046
10	Study design	clinical trial, phase iv.pt.	2,517
11	Study design	controlled clinical trial.pt.	95,575
12	Study design	randomized controlled trial.pt.	617,667
13	Study design	multicenter study.pt.	350,629
14	Study design	clinical trial.pt.	540,236
15	Study design	exp Clinical Trials as topic/	394,602
16	Study design	or/1-15	1,618,049
17	Study design	(clinical adj trial$).tw.	518,813
18	Study design	((singl$ or doubl$ or treb$ or tripl$) adj (blind$3 or mask$3)).tw.	206,633
19	Study design	PLACEBOS/	35,972
20	Study design	placebo$.tw.	258,185
21	Study design	randomly allocated.tw.	39,078
22	Study design	(allocated adj2 random$).tw.	43,026
23	Study design	or/17-22	835,386
24	Study design	16 or 23	1,998,048
25	Study design	case report.tw.	432,010
26	Study design	letter/	1,263,111
27	Study design	historical article/	370,775
28	Study design	or/25-27	2,045,493
29	Study design	24 not 28	1,953,742
30	Population	exp pneumonia, bacterial/	23,874
31	Population	(bacterial and pneumonia).mp.	48,005
32	Population	30 or 31	54,542
33	Population	((hospital or ventilator or nosocomial) and pneumonia).mp.	52,559
34	Population	32 and 33	13,896
35	Population	exp pneumonia, ventilator-associated/	4,475
36	Population	34 or 35	16,534
37	Intervention	meropenem/	3,911
38	Intervention	(meropenem or Merrem or Penem or Ronem or SM 7338 or SM- 7338).mp.	11,568
39	Intervention	(ceftolozane or 'CXA-201' or 'cb-500,201' or cb500201 or 'cxa- 201' or cxa201 or 'cxa 101 plus tazobactam' or 'ceftolozane tazobactam' or 'ceftolozane plus	948

Confidential - Not for Public Consumption or Distribution

No.	Criteria	Strings	Hits
		tazobactam' or 'ceftolozane-tazobactam' or (ceftolozane adj2 tazobactam)).mp.	
40	Intervention	PIPERACILLIN, TAZOBACTAM DRUG COMBINATION/	1,409
41	Intervention	('piperacillin plus tazobactam' or (Piperacillin and tazobactam) or (piperacillin adj2 tazobactam) or 'piperacillin-tazobactam' or Tazocin or Zosyn or tazopril or tazobac or tazonam or tazocillin or tazocilline or tazomax or tazocel).mp.	6,463
42	Intervention	cefepime/	1,664
43	Intervention	(cefepim or cefepime or cefepime hyrdrochloride or Quadrocef or Maxipime or Axepim or BMY 28142 or BMY-28142).ti,ab,kw.	4,743
44	Intervention	exp levofloxacin/	4,239
45	Intervention	(levofloxacin or Levaquin or Tavanic or iquix or mosardal or nofaxin or levox or 'cravit aphtalmic' or levokacin or lesacin or tavanic or quixin or cravit or reskuin or levaquin or floxel or leroxacin or volequin or elequine).mp.	10,899
46	Intervention	exp ceftazidime/	4,933
47	Intervention	(ceftazidime or Fortum or LY-139381 or LY 139381 or LY139381 or tazidime or Fortaz or GR-20263 or GR 20263 or GR20263).mp.	12,778
48	Intervention	exp imipenem/	4,676
49	Intervention	(imipenem or Primaxin or MK-0787 or "MK 0787" or MK0787 or N-Formimidoylthienamycin or N Formimidoylthienamycin).mp.	13,792
50	Intervention	ciprofloxacin/	15,631
51	Intervention	(ciprofloxacin or Ciloxan or Cipro or Neofloxin).mp.	36,101
52	Intervention	(prulifloxacin or Quisnon or Unidrox or Prixina or Glimbax).mp.	161
53	Intervention	ertapenem/	828
54	Intervention	(ertapenem or Invanz).mp.	2,271
55	Intervention	doripenem/	386
56	Intervention	(doripenem or Doribax or Finibax).mp.	826
57	Intervention	exp ceftriaxone/	7,049
58	Intervention	(ceftriaxone or Rocephin or Epicephin).mp.	16,309
59	Intervention	exp amikacin/	4,686
60	Intervention	(amikacin or Yectamid or Amikacina or Amikafur or amikalem or Amikason's or Amikin or Biklin or Biclin or Amiklin or Amukin or BB-K8 or BB K8 or BBK8 or BB-K 8 or BB K 8 or BBK 8 or gamikal or kanbine or oprad).mp.	12,779
61	Intervention	exp gentamicins/	20,247
62	Intervention	(gentamycin or garamycin or gentacyclo or Gentavet or genticin or G-Myticin or Gmyticin or G Myticin or Gentamicin or Gentamycin).mp.	31,939
63	Intervention	exp tobramycin/	4,708

Confidential - Not for Public Consumption or Distribution

No.	Criteria	Strings	Hits
64	Intervention	(tobramycin or obracin or tobracin or brulamycin or nebcin or nebicin or nebramycin).mp.	9,082
65	Intervention	(plazomicin or Zemdri).mp.	223
66	Intervention	('colistin plus polymyxin' or 'colistin-polymyxin' or (colistin adj2 polymyxin)).mp.	536
67	Intervention	exp colistin/	5,975
68	Intervention	("Colisticin" or "Polymyxin E" or "Colimycin" or "Coly-Mycin" or "Totazina" or "Colistin Sulfate" or "Sulfate, Colistin").mp.	978
69	Intervention	exp polymyxins/	11,807
70	Intervention	("Polymyxin" or "Polymyxin M").mp.	9,775
71	Intervention	cefiderocol/	379
72	Intervention	cefiderocol.mp.	807
73	Intervention	or/37-72	136,946
74	Intervention	29 and 36 and 73	531
75	Time period	(2018$ or 2019$ or 2020$ or 2021$ or 2022$ or 2023$ or 2024$).ez,ep,dt.	9,601,616
76	Time period	("2018" or "2019" or "2020" or "2021" or "2022" or "2023" or "2024").yr.	9,477,547
77	Combined	74 and (75 or 76)	158
78	Language	limit 77 to english language	157

Confidential - Not for Public Consumption or Distribution

Table S13. HABP/VABP Search strategy for Cochrane Database of Systematic Reviews, search executed July 18, 2024

No.	Criteria	Strings	Hits
1	Population	[mh "pneumonia, bacterial"]	1,048
2	Population	bacterial:ti,ab,kw AND pneumonia:ti,ab,kw	3,698
3	Population	#1 OR #2	3,866
4	Population	((hospital:ti,ab,kw OR ventilator:ti,ab,kw OR nosocomial:ti,ab,kw) AND pneumonia:ti,ab,kw)	7,796
5	Population	#3 AND #4	1,667
6	Population	[mh "pneumonia, ventilator-associated"]	630
7	Population	#5 OR #6	2,054
8	Intervention	[mh ^meropenem]	369
9	Intervention	meropenem:ti,ab,kw OR Merrem:ti,ab,kw OR Penem:ti,ab,kw OR Ronem:ti,ab,kw OR "SM 7338":ti,ab,kw	894
10	Intervention	ceftolozane:ti,ab,kw OR "CXA 201":ti,ab,kw OR "cb 500 201":ti,ab,kw OR cb500201:ti,ab,kw OR cxa201:ti,ab,kw OR "'cxa 101 plus tazobactam'":ti,ab,kw OR "ceftolozane tazobactam":ti,ab,kw OR "ceftolozane plus tazobactam":ti,ab,kw OR (ceftolozane NEAR/2 tazobactam):ti,ab,kw	84
11	Intervention	[mh ^"PIPERACILLIN, TAZOBACTAM DRUG COMBINATION"]	218
12	Intervention	piperacillin plus tazobactam:ti,ab,kw OR (Piperacillin:ti,ab,kw AND tazobactam:ti,ab,kw) OR (piperacillin NEAR/2 tazobactam):ti,ab,kw OR "piperacillin tazobactam":ti,ab,kw OR Tazocin:ti,ab,kw OR Zosyn:ti,ab,kw OR tazopril:ti,ab,kw OR tazobac:ti,ab,kw OR tazonam:ti,ab,kw OR tazocillin:ti,ab,kw OR tazocilline:ti,ab,kw OR tazomax:ti,ab,kw OR tazocel:ti,ab,kw	713
13	Intervention	[mh ^cefepime]	195
14	Intervention	cefepim:ti,ab,kw OR cefepime:ti,ab,kw OR "cefepime hyrdrochloride":ti,ab,kw OR Quadrocef:ti,ab,kw OR Maxipime:ti,ab,kw OR Axepim:ti,ab,kw OR "BMY 28142":ti,ab,kw	415
15	Intervention	[mh levofloxacin]	774
16	Intervention	levofloxacin:ti,ab,kw OR Levaquin:ti,ab,kw OR Tavanic:ti,ab,kw OR iquix:ti,ab,kw OR mosardal:ti,ab,kw OR nofaxin:ti,ab,kw OR levox:ti,ab,kw OR "'cravit aphtalmic'":ti,ab,kw OR levokacin:ti,ab,kw OR lesacin:ti,ab,kw OR tavanic:ti,ab,kw OR quixin:ti,ab,kw OR cravit:ti,ab,kw OR reskuin:ti,ab,kw OR levaquin:ti,ab,kw OR floxel:ti,ab,kw OR leroxacin:ti,ab,kw OR volequin:ti,ab,kw OR elequine:ti,ab,kw	1,899
17	Intervention	[mh ceftazidime]	562

Confidential - Not for Public Consumption or Distribution

No.	Criteria	Strings	Hits
18	Intervention	ceftazidime:ti,ab,kw OR Fortum:ti,ab,kw OR "LY 139381":ti,ab,kw OR LY139381:ti,ab,kw OR tazidime:ti,ab,kw OR Fortaz:ti,ab,kw OR "GR 20263":ti,ab,kw OR GR20263:ti,ab,kw	1,137
19	Intervention	[mh imipenem]	373
20	Intervention	imipenem:ti,ab,kw OR Primaxin:ti,ab,kw OR "MK 0787":ti,ab,kw OR MK0787:ti,ab,kw OR "N Formimidoylthienamycin":ti,ab,kw	766
21	Intervention	[mh ^ciprofloxacin]	1,400
22	Intervention	ciprofloxacin:ti,ab,kw OR Ciloxan:ti,ab,kw OR Cipro:ti,ab,kw OR Neofloxin:ti,ab,kw	3,011
23	Intervention	prulifloxacin:ti,ab,kw OR Quisnon:ti,ab,kw OR Unidrox:ti,ab,kw OR Prixina:ti,ab,kw OR Glimbax:ti,ab,kw	62
24	Intervention	[mh ^ertapenem]	146
25	Intervention	ertapenem:ti,ab,kw OR Invanz:ti,ab,kw	247
26	Intervention	[mh ^doripenem]	29
27	Intervention	doripenem:ti,ab,kw OR Doribax:ti,ab,kw OR Finibax:ti,ab,kw	95
28	Intervention	[mh ceftriaxone]	869
29	Intervention	ceftriaxone:ti,ab,kw OR Rocephin:ti,ab,kw OR Epicephin:ti,ab,kw	1,861
30	Intervention	[mh amikacin]	440
31	Intervention	amikacin:ti,ab,kw OR Yectamid:ti,ab,kw OR Amikacina:ti,ab,kw OR Amikafur:ti,ab,kw OR amikalem:ti,ab,kw OR "Amikason s":ti,ab,kw OR Amikin:ti,ab,kw OR Biklin:ti,ab,kw OR Biclin:ti,ab,kw OR Amiklin:ti,ab,kw OR Amukin:ti,ab,kw OR "BB K8":ti,ab,kw OR BBK8:ti,ab,kw OR "BB K 8":ti,ab,kw OR "BBK 8":ti,ab,kw OR gamikal:ti,ab,kw OR kanbine:ti,ab,kw OR oprad:ti,ab,kw	985
32	Intervention	[mh gentamicins]	1,428
33	Intervention	gentamycin:ti,ab,kw OR garamycin:ti,ab,kw OR gentacyclo:ti,ab,kw OR Gentavet:ti,ab,kw OR genticin:ti,ab,kw OR Gmyticin:ti,ab,kw OR "G Myticin":ti,ab,kw OR Gentamicin:ti,ab,kw OR Gentamycin:ti,ab,kw	2,269
34	Intervention	[mh tobramycin]	703
35	Intervention	tobramycin:ti,ab,kw OR obracin:ti,ab,kw OR tobracin:ti,ab,kw OR brulamycin:ti,ab,kw OR nebcin:ti,ab,kw OR nebicin:ti,ab,kw OR nebramycin:ti,ab,kw	1,561
36	Intervention	plazomicin:ti,ab,kw OR Zemdri:ti,ab,kw	22
37	Intervention	colistin plus polymyxin:ti,ab,kw OR "colistin polymyxin":ti,ab,kw OR (colistin NEAR/2 polymyxin):ti,ab,kw	10
38	Intervention	[mh colistin]	231
39	Intervention	Colisticin:ti,ab,kw OR "Polymyxin E":ti,ab,kw OR Colimycin:ti,ab,kw OR "Coly Mycin":ti,ab,kw OR	96

Confidential - Not for Public Consumption or Distribution

No.	Criteria	Strings	Hits
		Totazina:ti,ab,kw OR "Colistin Sulfate":ti,ab,kw OR "Sulfate Colistin":ti,ab,kw	
40	Intervention	[mh polymyxins]	505
41	Intervention	Polymyxin:ti,ab,kw OR "Polymyxin M":ti,ab,kw	485
42	Intervention	[mh ^cefiderocol]	16
43	Intervention	cefiderocol:ti,ab,kw	36
44	Intervention	28-#43	13,154
45	Time period	#7 AND #44 with Publication Year from 2018 to 2024, in Trials	162
46	Language	english:la	2,064,013
47	Combined	#45 AND #46	161

Confidential - Not for Public Consumption or Distribution

Table S14. All-cause mortality with C/T versus meropenem in patient subgroups with ventilated hospital-acquired bacterial pneumonia/ventilator-associated bacterial pneumonia (ASPECT-NP)

Study (Year)	Outcome	C/T (% [n/N])	Meropenem (% [n/N])	Percent Difference, % (95% CI)
Kollef (2019)[11]	VAP/vHAP (mITT)[a] (Subgroups by baseline susceptible LRT pathogens)			
	28-day ACM	20.1 (53/264)	25.5 (63/247)	4.4 (−2.8 to 11.8)
	VAP/vHAP (mITT)[a] (Subgroups by augmented renal clearance)			
	28-day ACM: By CrCl ≥150 mL/min	9.4 (5/53)	15 (6/40)	5.6 (−7.85 to 20.62)
	28-day ACM: By CrCl <150 mL/min	22.7 (48/211)	27.5 (57/207)	4.8 (−3.53 to 13.04)
	28-day ACM: By CrCl >130 mL/min	13 (10/77)	20 (14/70)	7.0 (−5.05 to 19.28)
	28-day ACM: By CrCl ≤130 mL/min	23 (43/187)	27.7 (49/177)	4.7 (−4.23 to 13.57)
Martin-Loeches (2022)[12]	VABP/vHABP (mITT)[a] (Subgroups by LRT pathogens)			
	28-day ACM: By pathogen Gram-negative pathogens	20.1 (52/259)	25.8 (62/240)	5.8 (−1.62 to 13.12)
	VABP/vHABP (mITT)[a] (Subgroups by LRT pathogens susceptible to treatment)			
	28-day ACM: By pathogen *Enterobacterales*	14.7 (22/150)	25.7 (44/171)	11.1 (2.21 to 19.57)
	28-day ACM: By pathogen ESBL-producing *Enterobacterales*	13 (6/46)	29.4 (20/68)	16.4 (0.57 to 29.96)
	28-day ACM: By pathogen *P. aeruginosa*	24.5 (13/53)	19.6 (10/51)	−4.9 (−20.54 to 11.12)
	vHABP(ITT)[b] (Subgroups by pathogen)			
	28-day ACM: By LRT pathogens susceptible to randomized study drug	18.4 (7/38)	36.4 (20/55)	17.9 (− 0.9 to 34.0)
	28-day ACM: By ≥ 1 LRT pathogen	27 (10/37)	42.3 (11/26)	15.3 (−7.9 to 37.3)
Martin-Loeches (2023)[14]	vHABP/VABP (ITT)[b] (Subgroups by respiratory or cardiovascular dysfunction)			
	28-day ACM: By R-SOFA ≥ 2 + CV-SOFA ≥ 2	34.8 (24/NR)	30.8 (28/NR)	−4.0 (−18.6 to 10.3)
	28-day ACM: By R-SOFA ≥ 2	23.7 (74/312)	24.0 (77/321)	0.3 (−6.4 to 6.9)

Confidential - Not for Public Consumption or Distribution

Study (Year)	Outcome	C/T (% [n/N])	Meropenem (% [n/N])	Percent Difference, % (95% CI)
	28-day ACM: By CV-SOFA ≥ 2	33.3 (28/84)	30.3 (30/99)	−3.0 (−16.4 to 10.3)
	28-day ACM: By R SOFA score ≥3	23 (45/196)	23 (46/200)	0 (−8.2 to 8.3)
	28-day ACM: By R SOFA score 4	33.3 (8/24)	37.9 (11/29)	4.6 (−20.5 to 28.3)
	28-day ACM: By CV SOFA score ≥3	35.1 (27/77)	30.4 (28/92)	−4.6 (−18.6 to 9.3)
	28-day ACM: By CV SOFA score 4	41.9 (13/31)	34.1 (14/41)	−7.8 (−29.2 to 14)
	28-day ACM: By SOFA ≤6	18.4 (38/207)	20.5 (38/185)	2.2 (−5.6 to 10.1)
	28-day ACM: By SOFA >6	31.6 (49/155)	29.9 (53/177)	−1.7 (−11.6 to 8.2)
Shorr (2021)[15]	vHABP/VABP (ITT)[b] (Subgroups by kidney functions)			
	28-day ACM: By normal RF*	17.6 (23/131)	20.3 (25/123)	NR
	28-day ACM: By ARC*	17.7 (17/96)	17.7 (20/113)	NR
Huntington (2020)[16]	vHABP/VABP (mITT)[c] (Subgroups by renal impairment)			
	28-day ACM: By normal RF**	13.8 (24/174)	19 (30/158)	5.2 (−2.8 to 13.3)
	28-day ACM: By mild RI**	34.5 (19/55)	30.4 (17/56)	−4.2 (−21.0 to 12.9)
	28-day ACM: By moderate RI**	27.3 (6/22)	42.1 (8/19)	14.8 (−13.4 to 40.7)
	28-day ACM: By severe RI**	30.8 (4/13)	57.1 (8/14)	26.4 (−10.0 to 54.5)
Kollef (2022)[17]	vHABP/VABP (mITT)[a] (Subgroup by failure of therapy)			
	28-day ACM	17.9 (7/39)	45.8 (11/24)	27.9 (4.7 to 49.0)
	vHABP/VABP (ME)[d] (Subgroup by failure of therapy)			
	28-day ACM	22.6 (12/53)	45.0 (18/40)	22.4 (3.1 to 40.1)
Paterson (2022)[18]	vHABP/VABP (ITT)[b] (Subgroups by baseline susceptible LRT pathogens)			
	28-day ACM: Overall	6.7 (2/30)	32.3 (10/31)	25.6 (5.54 to 43.84)
	vHABP/VABP (ITT)[b] (Subgroups by *Enterobacterales* LRT isolates at baseline (≥1 ESBL-positive)			
	28-day ACM: Overall	8.0 (2/25)	33.3 (8/24)	25.3 (2.45 to 46.11)

Confidential - Not for Public Consumption or Distribution

*Abbreviations: ARC: Augmented Renal Clearance; CI: Confidence Interval; CE: Clinical Evaluable; CrCL: Creatinine Clearance; CV-SOFA: Cardiovascular-Sequential Organ Failure Assessment; ESBL: Extended Spectrum Beta-Lactamase; ITT: Intention-to-Treat; LRT: Lower respiratory tract; MDR: Multidrug-Resistant; ME: Microbiological evaluable; mITT: Microbiological Intention-to-Treat Population; NR: Not Reported; P. aeruginosa: Pseudomonas aeruginosa; RF: Renal Function; RI: Renal Impairment; R-SOFA: Respiratory-Sequential Organ Failure Assessment; SOFA: Sequential Organ Failure Assessment; TOC: Test-of-Cure; VABP: Ventilator-Associated Bacterial Pneumonia; vHABP: Ventilated Hospital-Associated Bacterial Pneumonia. a mITT was defined as the ITT participants who received ≥1 dose of study treatment and with ≥1 Gram-negative or streptococcal respiratory pathogen from baseline lower respiratory tract (LRT) cultures confirmed to be susceptible to ≥1 study drug. b ITT was defined as all randomized participants, regardless of whether they received study drug. c mITT population was defined as a subset of the ITT population who receive any amount of study drug and have at least one bacterial respiratory pathogen isolated from the baseline lower respiratory tract (LRT) culture that is susceptible to at least one of the study drugs.d Microbiologically evaluable (ME) population was defined as participants who received study drug, adhered to protocol requirements, had ≥ 1 Gram-negative or streptococcal respiratory pathogen from baseline lower respiratory tract (LRT) cultures. * Normal renal function, CrCl: ≥80 to 130mL/min, augmented renal clearance, CrCl >130mL/min. ** Normal renal function (CrCl, ≥80 ml/min), mild RI (CrCl, >50 to <80 ml/min), moderate RI (CrCl, ≥30 to ≤50 ml/min), severe RI (CrCl, ≥15 to <30 ml/min).*

Confidential - Not for Public Consumption or Distribution

Table S15. Rates of clinical cure with C/T versus meropenem for patients subgroups with ventilated hospital-acquired bacterial pneumonia/ventilator-associated bacterial pneumonia (ASPECT-NP).

Study (Year)	Outcome	C/T (% [n/N])	Meropenem (% [n/N])	Percent Difference, % (95% CI)
Kollef (2019)[11]	VAP/vHAP (mITT)[a] (Subgroups by baseline susceptible LRT pathogens)			
	Per-patient clinical cure at TOC	64.8 (140/216)	56.5 (118/209)	7.1 (−2.24 to 16.26)
	Per-patient clinical cure at TOC: By Gram-negative pathogen	64.7 (139/215)	56.4 (115/204)	8.3 (−1.07 to 17.45)
	Per-patient clinical cure at TOC: By *Enterobacteriaceae*	64.0 (103/161)	55.4 (87/157)	8.6 (−2.20 to 19.06)
	Per-patient clinical cure at TOC: By ESBL-producing *Enterobacteriaceae*	62.3 (33/53)	62.5 (30/48)	−0.2 (−18.46 to 18.18)
	Per-patient clinical cure at TOC: By *P. aeruginosa*	61.7 (29/47)	60.7 (34/56)	1.0 (−17.47 to 19.09)
	vHAP/VAP (CE)[b] (Subgroups by augmented renal clearance)			
	Clinical cure at TOC: By CrCl ≥150 mL/min	56.1 (23/41)	67.4 (29/41)	−11.3 (−30.64 to 9.13)
	Clinical cure at TOC: By CrCl <150 mL/min	65.5 (116/177)	64.0 (114/178)	1.5 (−8.38 to 11.32)
	Clinical cure at TOC: By CrCl >130 mL/min	59.7 (37/62)	67.6 (46/68)	−8.0 (−23.87 to 8.37)
	Clinical cure at TOC: By CrCl ≤130 mL/min	65.4 (102/156)	63.4 (97/153)	2.0 (−8.61 to 12.53)
Martin-Loeches (2022)[12]	VABP/vHABP (mITT)[a] (Subgroups by LRT pathogens susceptible to treatment)			
	Clinical cure at TOC: By *Enterobacterales*	66.0 (99/NR)	57.3 (98/NR)	8.7 (−1.98 to 19.01)
	Clinical cure at TOC: By ESBL-producing *Enterobacterales*	65.2 (30/NR)	61.8 (42/NR)	3.5 (−14.48 to 20.14)
	Clinical cure at TOC: By *P. aeruginosa*	58.5 (31/NR)	58.8 (30/NR)	−0.3 (−18.60 to 18.01)
	VABP/vHABP (ME)[c] (Subgroups by LRT pathogens)			
	Clinical cure at TOC: Gram-negative pathogens	75.2 (85/NR)	66.7 (78/NR)	8.6 (−3.19 to 19.94)

Confidential - Not for Public Consumption or Distribution

Study (Year)	Outcome	C/T (% [n/N])	Meropenem (% [n/N])	Percent Difference, % (95% CI)
Martin-Loeches (2023)[14]	vHABP/VABP (ITT)[d] (Subgroups by respiratory or cardiovascular dysfunction)			
	Clinical cure: By R-SOFA ≥ 2 + CV-SOFA ≥ 2	53.6 (37/69)	56 (51/91)	−2.4 (−17.6 to 12.8)
	Clinical cure: By R-SOFA ≥ 2	55.8 (174/312)	54.2 (174/321)	1.6 (−6.2 to 9.3)
	Clinical cure: By CV-SOFA ≥ 2	53.6 (45/84)	55.6 (55/99)	−2.0 (−16.1 to 12.2)
	Clinical cure: By R SOFA score ≥3	59.7 (117/1966)	55.5 (111/200)	4.2 (−5.5 to 13.8)
	Clinical cure: By R SOFA score 4	62.5 (15/24)	44.8 (13/29)	17.7 (− 8.8 to 40.8)
	Clinical cure: By CV SOFA score ≥3	54.5 (42/77)	55.4 (51/92)	−0.9 (−15.6 to 13.8)
	Clinical cure: By CV SOFA score 4	51.6 (16/31)	53.7 (22/41)	−2.1 (−24.1 to 20.1)
	Clinical cure: By SOFA ≤6	56.5 (117/207)	54.6 (101/185)	1.9 (−7.9 to 11.7)
	Clinical cure: By SOFA >6	51.6 (80/155)	52.5 (93/177)	−0.9 (−11.6 to 9.7)
Shorr (2021)[15]	vHABP/VABP (ITT)[d] (Subgroups by kidney functions)			
	Clinical cure at TOC: By normal RF*	57.3 (75/131)	59.3 (73/123)	NR
	Clinical cure at TOC: By ARC*	59.4 (57/96)	57.5 (65/113)	NR
Huntington (2020)[16]	vHABP/VABP (ITT)[d] (Subgroups by renal impairment)			
	Clinical cure: By normal RF**	58.2 (132/NR)	58.5 (138/NR)	−0.3 (−9.2 to 8.6)
	Clinical cure: By mild RI**	54.9 (45)	45.5 (35)	9.4 (−6.0 to 24.2)
	Clinical cure: By moderate RI**	37.1 (13/NR)	42.3 (11/NR)	−5.2 (−28.6 to 18.4)
	Clinical cure: By severe RI**	41.2 (7/NR)	47.6 (10/NR)	−6.4 (−34.4 to 23.4)
Kollef (2022)[17]	vHABP/VABP (ITT)[d] (Subgroup by failure of therapy)			
	Clinical cure at TOC: By failure of initial anti-bacterial therapy	49.1 (26/53)	37.5 (15/40)	11.6 (−8.6 to 30.2)
	vHABP/VABP (CE)[b] (Subgroup by failure of therapy)			

Confidential - Not for Public Consumption or Distribution

Study (Year)	Outcome	C/T (% [n/N])	Meropenem (% [n/N])	Percent Difference, % (95% CI)
	Clinical cure at TOC: By failure of initial anti-bacterial therapy	63.6 (21/33)	45.0 (9/20)	18.6 (−8.2 to 42.5)
Paterson (2022)[18]	vHABP/VABP (ITT)[d] (Subgroups by baseline susceptible LRT pathogens)			
	Clinical cure at TOC: Overall	73.3 (22/NR)	61.3 (19/NR)	12.0 (−11.21 to 33.51)
	vHABP/VABP (ITT)[d] (Subgroups by *Enterobacterales* LRT isolates at baseline (≥1 ESBL-positive)			
	Clinical cure at TOC: Overall	72.0 (18/25)	66.7 (16/24)	5.3 (−19.55 to 29.55)
Martin-Loeches (2019)[19]	vHABP/VABP (mITT)[a] (Subgroups by baseline LRT pathogen susceptible to ≥1 study drug)			
	Clinical cure: Overall	60.6 (157/259)	57.1 (137/240)	3.5 (−5.07 to 12.08)

*Abbreviations: ARC: Augmented Renal Clearance; CI: Confidence Interval; CE: Clinical Evaluable; CrCL: Creatinine Clearance; CV-SOFA: Cardiovascular-Sequential Organ Failure Assessment; ESBL: Extended Spectrum Beta-Lactamase; ITT: Intention-to-Treat; LRT: Lower respiratory tract; ME: Microbiological Evaluable; mITT: Microbiological Intention-to-Treat Population; NR: Not Reported; P. aeruginosa: Pseudomonas aeruginosa; RF: Renal Function; RI: Renal Impairment; R-SOFA: Respiratory-Sequential Organ Failure Assessment; SOFA: Sequential Organ Failure Assessment; TOC: Test-of-Cure; VABP: Ventilator-Associated Bacterial Pneumonia; vHABP: Ventilated Hospital-Associated Bacterial Pneumonia. a mITT was defined as the ITT participants who received ≥1 dose of study treatment and with ≥1 Gram-negative or streptococcal respiratory pathogen from baseline lower respiratory tract (LRT) cultures confirmed to be susceptible to ≥1 study drug. b CE Clinically evaluable (CE) population was defined as the participants received study drug, adhered to the study protocol through the test-of-cure (TOC) visit, and had an evaluable clinical outcome at the TOC visit or clinical failure prior to the TOC visit. c Microbiologically evaluable (ME) population was defined as participants who received study drug, adhered to protocol requirements, had ≥ 1 Gram-negative or streptococcal respiratory pathogen from baseline lower respiratory tract (LRT) cultures. d ITT was defined as all randomized participants, regardless of whether they received study drug. * Normal renal function, CrCl: ≥80 to 130mL/min, augmented renal clearance, CrCl >130mL/min. ** Normal renal function (CrCl, ≥80 ml/min), mild RI (CrCl, >50 to <80 ml/min), moderate RI (CrCl, ≥30 to ≤50 ml/min), severe RI (CrCl, ≥15 to <30 ml/min).*

Confidential - Not for Public Consumption or Distribution

Table S16. Rates of clinical cure with C/T + metronidazole versus meropenem in patient subgroups with complex intra-abdominal infection (ASPECT-cIAI).

Study (Year)	Efficacy	C/T + Metronidazole (% [n/N])	Meropenem (% [n/N])	Percent Difference, % (95% CI)
**ASPECT-cIAI**				
Solomkin (2015)[20]	cIAI (ME)[a] (Subgroups by renal impairment)			
	Clinical cure at the TOC visit: Moderate renal impairment CrCl <50 mL/min	72.7 (8/11)	71.4 (5/7)	1.3 (−34.37 to 40.92)
	Clinical cure at the TOC visit: Mild renal impairment CrCl ≥50 mL/min	95.1 (251/264)	95.2 (299/314)	−0.1 (−3.95 to 3.43)
Miller et al. 2015[22]	cIAI (CE)[b] (Subgroup by European adult patients)			
	Clinical cure at TOC visit for subgroup: By European adult patients	95.4 (NR)	95.1 (NR)	0.6 (−3.99 to 5.13)
	cIAI (ME)[a] (European adult patients, subgroups by pathogens)			
	Clinical cure at TOC for subgroup: By European adult patients with Gram negative aerobes	85.9 (213/248)	89.6 (240/268)	NR
	Clinical cure at TOC for subgroup: By European adult patients with ESBL producing *E.coli*	100 (14/14)	100 (14/14)	NR
	Clinical cure at TOC for subgroup: By European adult patients with ESBL producing *Klebsiella pneumonia*	100 (6/6)	75 (3/4)	NR
	Clinical cure at TOC for subgroup: By European adult patients with *P.aeruginosa*	100 (16/16)	95.8 (23/24)	NR
Miller et al. 2016[21]	cIAI (ME)[a] (Subgroups by pathogens)			
	Clinical cure for Adult patients with baseline *P.aeruginosa*	100 (26/26)	93.1 (27/29)	NR

Confidential - Not for Public Consumption or Distribution

Study (Year)	Efficacy	C/T + Metronidazole (% [n/N])	Meropenem (% [n/N])	Percent Difference, % (95% CI)
	Clinical cure for Adult patients without baseline *P.aeruginosa*	93.2 (262/281)	93 (294/316)	NR
**NCT01147640**				
Lucasti. (2014)[23]	cIAI (ME)[a] (Subgroups by renal function)			
	Clinical cure for subgroup: By normal renal function (≥90 ml/min/1.73m^2)	87.9 (29/33)	NR	NR
	Clinical cure for subgroup: By mild to moderate renal impairment (60 to <90 ml/min/1.73m^2)	90 (18/20)	NR	NR

Abbreviations: CE: Clinical Evaluable; CI: Confidence Interval; cIAI: Complicated Intra-Abdominal Infection; CrCL: Creatinine Clearance; ME: Microbiologically Evaluable; min: Minute; ml: milliliter; NR: Not reported; TOC: Test-of-cure. a The microbiologically evaluable (ME) population was defined as the patients in the clinical evaluable (CE) population who had an intra-abdominal pathogen at baseline that was susceptible to the study drug received.b Clinically evaluable (CE) population was defined as the participants received study drug, adhered to the study protocol through the test-of-cure (TOC) visit, and had an evaluable clinical outcome at the TOC visit or clinical failure prior to the TOC visit.

Confidential - Not for Public Consumption or Distribution

Table S17. Rates of microbiological eradication with C/T versus meropenem for patient subgroups with ventilated hospital-acquired bacterial pneumonia/ventilator-associated bacterial pneumonia (ASPECT-NP).

Study (Year)	Outcome	C/T (% [n/N])	Meropenem (% [n/N])	Percent Difference, % (95% CI)
Kollef (2019)[11]	VABP/vHABP (mITT)[a] (Subgroups by baseline susceptible LRT pathogens)			
	Microbiological eradication at TOC	73.1 (193/264)	68.0 (168/247)	4.5 (−3.4 to 12.5)
Martin-Loeches (2022)[12]	VABP/vHABP (ME)[b] (Subgroups by LRT pathogens)			
	Microbiological eradication at TOC: By Gram-negative pathogens (overall)	69.9 (79/NR)	62.4 (73/NR)	7.5 (−4.69 to 19.38)
Martin-Loeches (2023)[14]	vHABP/VABP (mITT)[a] (Subgroups by respiratory or cardiovascular dysfunction)			
	Microbiological eradication: By R-SOFA ≥ 2 + CV-SOFA ≥ 2	47.8 (NR)	25.8 (NR)	17.5 (0.6 to 32.1)
	Microbiological eradication: By R-SOFA ≥ 2	30.2 (NR)	27.5 (NR)	6.6 (−1.7 to 14.9)
	Microbiological eradication: By CV-SOFA ≥ 2	44.6 (NR)	26.4 (NR)	16.5 (0.6 to 30.7)
	Microbiological/presumed eradication: By SOFA ≤6	73.2 (115/157)	69.7 (85/122)	3.6 (−6.9 to 14.3)
	Microbiological/presumed eradication: By SOFA >6	72.9 (78/107)	66.4 (83/125)	6.5 (−5.4 to 18)
Shorr (2021)[15]	vHABP/VABP (mITT)[b] (Subgroups by kidney functions)			
	Per-patient microbiological cure: By normal RF*	72.2 (70/97)	75 (66/88)	NR
	Per-patient microbiological cure: By ARC*	71.4 (55/77)	70 (49/70)	NR
Huntington (2020)[16]	vHABP/VABP (mITT)[a] (Subgroups by renal impairment)			
	Microbiological cure or presumed: By normal RF**	71.8 (125/174)	72.8 (115/158)	−0.9 (−10.5 to 8.7)

Confidential - Not for Public Consumption or Distribution

Study (Year)	Outcome	C/T (% [n/N])	Meropenem (% [n/N])	Percent Difference, % (95% CI)
	Microbiological cure or presumed: By mild RI**	83.6 (46/55)	57.1 (32/56)	26.5 (9.5 to 41.5)
	Microbiological cure or presumed: By moderate RI**	59.1 (13/22)	68.4 (13/19)	−9.3 (−35.4 to 19.2)
	Microbiological cure or presumed: By severe RI**	69.2 (9/13)	57.1 (8/14)	12.1 (−22.3 to 42.6)
Kollef (2022)[17]	vHABP/VABP (mITT)[a] (Subgroup by failure of therapy)			
	Microbiological eradication at TOC: By failure of initial anti-bacterial therapy	66.7 (26/39)	66.7 (16/24)	0.0 (−21.96 to 23.66)
Martin-Loeches (2019)[19]	vHABP/VABP (mITT)[a] (Subgroups by LRT pathogens)			
	Microbiological eradication: By pathogen *Enterobacterales*	74.4 (145/195)	74.4 (129/185)	4.6 (−4.37 to 13.58)
	Microbiological eradication: By pathogen *E. Coli*	84.3 (43/51)	78.6 (33/42)	5.7 (−9.96 to 22.08)
	Microbiological eradication: By pathogen *K. Pneumoniae*	73.3 (63/86)	71.4 (65/91)	1.8 (−11.30 to 14.77)
	Microbiological eradication: By pathogen ESBL-producing *Enterobacterales*	66.7 (56/84)	71.2 (52/73)	−4.6 (−18.56 to 9.93)
	Microbiological eradication: By pathogen *P. aeruginosa*	74.6 (47/63)	63.1 (41/65)	11.5 (−4.51 to 26.72)

Abbreviations: ARC: Augmented Renal Clearance; CI: Confidence Interval; CE: Clinical Evaluable; CrCL: Creatinine Clearance; CV-SOFA: Cardiovascular-Sequential Organ Failure Assessment; E. coli: Escherichia coli; ESBL: Extended Spectrum Beta-Lactamase; ITT: intention-to-treat; K. pneumoniae: Klebsiella pneumoniae; LRT: Lower Respiratory Tract; ME: Microbiological Evaluable; mITT: Microbiological Intention-to-Treat Population; NR: Not Reported; P. aeruginosa: Pseudomonas aeruginosa; RF: Renal Function; RI: Renal Impairment; R-SOFA: Respiratory-Sequential Organ Failure Assessment; SOFA: Sequential Organ Failure Assessment; TOC: Test-of-Cure; VABP: Ventilator-Associated Bacterial Pneumonia; vHABP: Ventilated Hospital-Acquired Bacterial Pneumonia. a mITT was defined as the ITT participants who received ≥1 dose of study treatment and with ≥1 Gram-negative or streptococcal respiratory pathogen from baseline lower respiratory tract (LRT) cultures confirmed to be susceptible to ≥1 study drug. b Microbiologically evaluable (ME) population was defined as participants who received study drug, adhered to protocol requirements, had ≥ 1 Gram-negative or streptococcal respiratory pathogen from baseline lower respiratory tract (LRT) cultures Normal renal function, CrCl: ≥80 to 130mL/min, augmented renal clearance, CrCl >130mL/min. ***

Confidential - Not for Public Consumption or Distribution

Normal renal function (CrCl, ≥80 ml/min), mild RI (CrCl, >50 to <80 ml/min), moderate RI (CrCl, ≥30 to ≤50 ml/min), severe RI (CrCl, ≥15 to <30 ml/min).

Table S18. PRISMA Checklist

Section and Topic	Item #	Checklist item	Location where item is reported
**TITLE**			
Title	1	Identify the report as a systematic review.	Manuscript — Title page ("…systematic reviews of comparative efficacy studies")
**ABSTRACT**			
Abstract	2	See the PRISMA 2020 for Abstracts checklist.	Manuscript — Abstract section
**INTRODUCTION**			
Rationale	3	Describe the rationale for the review in the context of existing knowledge.	Manuscript — Section 1. Introduction
Objectives	4	Provide an explicit statement of the objective(s) or question(s) the review addresses.	Manuscript — Section 1. Introduction
**METHODS**			
Eligibility criteria	5	Specify the inclusion and exclusion criteria for the review and how studies were grouped for the syntheses.	Manuscript — Section 2. Materials and Methods; PICOTS
Information sources	6	Specify all databases, registers, websites, organisations, reference lists and other sources searched or consulted to identify studies. Specify the date when each source was last searched or consulted.	Manuscript — Section 2. Materials and Methods; Data sources and search dates; Table S3 (databases); Tables S4–S13 (hand-search/conference sources) referenced
Search strategy	7	Present the full search strategies for all databases, registers and websites, including any filters and limits used.	Manuscript — Supplementary Tables S4–S13 (full strategies referenced)
Selection process	8	Specify the methods used to decide whether a study met the inclusion criteria of the review, including how many reviewers screened each record and each report retrieved, whether they worked independently, and if applicable, details of automation tools used in the process.	Manuscript — Section 2. Materials and Methods > Study selection (two reviewers; third adjudicator)
Data collection process	9	Specify the methods used to collect data from reports, including how many reviewers collected data from each report, whether they worked independently, any processes for obtaining or confirming data from study investigators, and if applicable, details of automation tools used in the process.	Manuscript — Section 2. Materials and Methods (data extraction described at high level; dual review implied)

Confidential - Not for Public Consumption or Distribution

Section and Topic	Item #	Checklist item	Location where item is reported
Data items	10a	List and define all outcomes for which data were sought. Specify whether all results that were compatible with each outcome domain in each study were sought (e.g. for all measures, time points, analyses), and if not, the methods used to decide which results to collect.	Manuscript — Section 2. Materials and Methods > Outcomes and definitions (ACM, clinical cure, microbiological eradication)
	10b	List and define all other variables for which data were sought (e.g. participant and intervention characteristics, funding sources). Describe any assumptions made about any missing or unclear information.	Manuscript — Section 2. Materials and Methods; Tables 1–2 (study and patient characteristics); Supplementary Tables S1–S3
Study risk of bias assessment	11	Specify the methods used to assess risk of bias in the included studies, including details of the tool(s) used, how many reviewers assessed each study and whether they worked independently, and if applicable, details of automation tools used in the process.	Manuscript — Section 2. Materials and Methods — Risk of bias (Cochrane RoB-2; dual assessment); Supplement — Risk of bias table/figure
Effect measures	12	Specify for each outcome the effect measure(s) (e.g. risk ratio, mean difference) used in the synthesis or presentation of results.	Manuscript — Results sections 3.2–3.4 and associated tables
Synthesis methods	13a	Describe the processes used to decide which studies were eligible for each synthesis (e.g. tabulating the study intervention characteristics and comparing against the planned groups for each synthesis (item #5)).	Manuscript — Section 2. Materials and Methods (grouping by indication; HABP/VABP vs cIAI/cUTI); Section 3. Results (per-indication syntheses)
	13b	Describe any methods required to prepare the data for presentation or synthesis, such as handling of missing summary statistics, or data conversions.	Methods 2. Materials and Methods. Results
	13c	Describe any methods used to tabulate or visually display results of individual studies and syntheses.	Manuscript — Section 2. Materials and Methods — Presentation of results; Section 3. Results — Structured tables/figures
	13d	Describe any methods used to synthesize results and provide a rationale for the choice(s). If meta-analysis was performed, describe the model(s), method(s) to identify the presence and extent of statistical	Manuscript — Section 2. Materials and Methods — Statistical synthesis and heterogeneity (narrative synthesis; no meta-analysis;

Confidential - Not for Public Consumption or Distribution

Section and Topic	Item #	Checklist item	Location where item is reported
		heterogeneity, and software package(s) used.	rationale stated; no heterogeneity statistics/software)
	13e	Describe any methods used to explore possible causes of heterogeneity among study results (e.g. subgroup analysis, meta-regression).	Manuscript — Section 2. Materials and Methods — Subgroup/heterogeneity assessment (pre-specified subgroups); Section 3. Results — Subgroup findings
	13f	Describe any sensitivity analyses conducted to assess robustness of the synthesized results.	Manuscript — Section 2. Materials and Methods — Sensitivity analyses (none planned/performed due to limited trials)
Reporting bias assessment	14	Describe any methods used to assess risk of bias due to missing results in a synthesis (arising from reporting biases).	Manuscript — Section 2. Materials and Methods — Reporting/publication bias assessment (not feasible; mitigation via conference and registry searches)
Certainty assessment	15	Describe any methods used to assess certainty (or confidence) in the body of evidence for an outcome.	Manuscript — Section 2. Materials and Methods — Certainty of evidence (no formal GRADE; narrative confidence)
**RESULTS**			
Study selection	16a	Describe the results of the search and selection process, from the number of records identified in the search to the number of studies included in the review, ideally using a flow diagram.	Manuscript — Section 3. Results — Study selection; Figures — PRISMA flow diagrams (HABP/VABP; cIAI/cUTI) with counts
	16b	Cite studies that might appear to meet the inclusion criteria, but which were excluded, and explain why they were excluded.	Manuscript — Section 3. Results — Excluded studies (reasons summarized); Supplement — Excluded studies table
Study characteristics	17	Cite each included study and present its characteristics.	Manuscript — Section 3. Results — Study characteristics; Tables 1–2 (designs, populations, interventions/comparators)
Risk of bias in studies	18	Present assessments of risk of bias for each included study.	Manuscript — Section 3. Results — Risk of bias; Supplement — RoB table/figure per study
Results of individual studies	19	For all outcomes, present, for each study: (a) summary statistics for each group (where appropriate) and (b) an effect estimate and its precision (e.g. confidence/credible interval), ideally using structured tables or plots.	Manuscript — Section 3. Results — Study-level outcomes; Tables (n/N, %, effect estimates, 95% CIs)
Results of syntheses	20a	For each synthesis, briefly summarise the characteristics and risk of bias among contributing studies.	Manuscript — Section 3. Results — Synthesis overview (characteristics and RoB of

Confidential - Not for Public Consumption or Distribution

Section and Topic	Item #	Checklist item	Location where item is reported
			contributing studies); Discussion — Evidence profile
	20b	Present results of all statistical syntheses conducted. If meta-analysis was done, present for each the summary estimate and its precision (e.g. confidence/credible interval) and measures of statistical heterogeneity. If comparing groups, describe the direction of the effect.	Manuscript — Section 3. Results — Statistical syntheses (no pooling; direction of effects described); Methods cross-reference
	20c	Present results of all investigations of possible causes of heterogeneity among study results.	Manuscript — Section 3. Results — Subgroup analyses/heterogeneity findings
	20d	Present results of all sensitivity analyses conducted to assess the robustness of the synthesized results.	Manuscript — Section 3. Results — Sensitivity analyses (none)
Reporting biases	21	Present assessments of risk of bias due to missing results (arising from reporting biases) for each synthesis assessed.	Manuscript — Section 3. Results — Reporting/publication bias (not assessed; rationale)
Certainty of evidence	22	Present assessments of certainty (or confidence) in the body of evidence for each outcome assessed.	Manuscript — Section 3. Results — Certainty of evidence summary (narrative)
**DISCUSSION**			
Discussion	23a	Provide a general interpretation of the results in the context of other evidence.	Manuscript — Section 4. Discussion — Interpretation in context of other evidence
	23b	Discuss any limitations of the evidence included in the review.	Manuscript — Section 6. Limitations — Evidence limitations
	23c	Discuss any limitations of the review processes used.	Manuscript — Section 6. Limitations — Methods/process limitations
	23d	Discuss implications of the results for practice, policy, and future research.	Manuscript — Section 4. Discussion and Conclusions — Implications for practice/policy/research
**OTHER INFORMATION**			
Registration and protocol	24a	Provide registration information for the review, including register name and registration number, or state that the review was not registered.	Manuscript — Section 2. Materials and Methods — Registration (statement of registration status)
	24b	Indicate where the review protocol can be accessed, or state that a protocol was not prepared.	Manuscript — Section 2. Materials and Methods — Protocol (access or "not prepared" statement)
	24c	Describe and explain any amendments to information	Manuscript — Section 2. Materials and Methods —

Confidential - Not for Public Consumption or Distribution

Section and Topic	Item #	Checklist item	Location where item is reported
		provided at registration or in the protocol.	Protocol amendments (if any; otherwise “not applicable”)
Support	25	Describe sources of financial or non-financial support for the review, and the role of the funders or sponsors in the review.	Manuscript — Funding section; Acknowledgments (role of funder/sponsor)
Competing interests	26	Declare any competing interests of review authors.	Manuscript — Conflicts of interest
Availability of data, code and other materials	27	Report which of the following are publicly available and where they can be found: template data collection forms; data extracted from included studies; data used for all analyses; analytic code; any other materials used in the review.	Manuscript — Data availability statement; Supplement — search strategies, PRISMA flows, extraction templates

Confidential - Not for Public Consumption or Distribution
